# Enhancing self-management in type 1 diabetes with wearables and deep learning

**DOI:** 10.1038/s41746-022-00626-5

**Published:** 2022-06-27

**Authors:** Taiyu Zhu, Chukwuma Uduku, Kezhi Li, Pau Herrero, Nick Oliver, Pantelis Georgiou

**Affiliations:** 1 Centre for Bio-Inspired Technology, Department of Electrical and Electronic Engineering, Imperial College London, London, UK; 2 Division of Diabetes, Endocrinology and Metabolism, Faculty of Medicine, Imperial College London, London, UK; 3 Institute of Health Informatics, University College London, London, UK

**Keywords:** Diagnosis, Type 1 diabetes

## Abstract

People living with type 1 diabetes (T1D) require lifelong self-management to maintain glucose levels in a safe range. Failure to do so can lead to adverse glycemic events with short and long-term complications. Continuous glucose monitoring (CGM) is widely used in T1D self-management for real-time glucose measurements, while smartphone apps are adopted as basic electronic diaries, data visualization tools, and simple decision support tools for insulin dosing. Applying a mixed effects logistic regression analysis to the outcomes of a six-week longitudinal study in 12 T1D adults using CGM and a clinically validated wearable sensor wristband (NCT ID: NCT03643692), we identified several significant associations between physiological measurements and hypo- and hyperglycemic events measured an hour later. We proceeded to develop a new smartphone-based platform, ARISES (Adaptive, Real-time, and Intelligent System to Enhance Self-care), with an embedded deep learning algorithm utilizing multi-modal data from CGM, daily entries of meal and bolus insulin, and the sensor wristband to predict glucose levels and hypo- and hyperglycemia. For a 60-minute prediction horizon, the proposed algorithm achieved the average root mean square error (RMSE) of 35.28 ± 5.77 mg/dL with the Matthews correlation coefficients for detecting hypoglycemia and hyperglycemia of 0.56 ± 0.07 and 0.70 ± 0.05, respectively. The use of wristband data significantly reduced the RMSE by 2.25 mg/dL (*p* < 0.01). The well-trained model is implemented on the ARISES app to provide real-time decision support. These results indicate that the ARISES has great potential to mitigate the risk of severe complications and enhance self-management for people with T1D.

## Introduction

Diabetes is a group of chronic metabolic disorders that affect almost half a billion people worldwide^1^, and around 10% of them have type 1 diabetes (T1D)^2^. Due to an absolute deficiency of endogenous insulin caused by pancreatic *β*-cell loss, the management of T1D relies on exogenous insulin delivery and adherence to a group of self-care behaviors, such as estimating dietary carbohydrate and exercise, and titrating insulin therapy. The primary objective of T1D self-management is to prevent immediate adverse glycemic events, including hypoglycemia and hyperglycemia, and minimize the risk of long-term diabetes complications. Severe hypoglycemia may cause seizures, brain damage, and intellectual impairment^3^, while hyperglycemia is a risk factor for cardiovascular diseases, neuropathy, nephropathy and retinopathy ^4^.

The development of continuous glucose monitoring (CGM) has led to therapeutic benefits in diabetes management^5,6^. The usage of real-time CGM systems has been demonstrated to reduce the number of severe hypoglycemic events for T1D subjects with multiple daily injection (MDI)^7^. As a wearable device that automatically measures glucose levels with a fixed frequency (e.g. five minutes), CGM can be combined with an insulin pump as sensor-augmented therapy or an artificial pancreas for closed-loop glycemic control^8,9^. Smartphone apps to log daily events^10,11^ and calculate bolus insulin are increasingly being adopted to successfully reduce the daily burden associated with T1D self-management. Other wearables, such as wristbands, have been used in recent literature to estimate physical activity for T1D subjects^12,13^. Nonetheless, the clinical efficacy of apps and sensor wristbands remains unproven^14^, and there is a lack of an integrated platform that synchronizes the real-time physiological measurements of sensor wristbands and other wearable devices to improve decision support^14,15^.

Despite CGM enabling correction of glucose concentrations outside of the target range ([70, 180] mg/dL), self-management can be challenging for people with T1D due to the variable pharmacokinetics and pharmacodynamics of insulin^16^ and the multiple endogenous and exogenous influences on glucose. Combined with CGM systems, a predictive low-glucose suspend feature, commonly found in continuous subcutaneous insulin infusion (CSII) systems, has been shown to significantly reduce exposure to hypoglycemia^17^. Accurate glucose prediction is, therefore, a useful tool to enable proactive interventions and timely medication administration to enhance T1D self-management. However, the performance of physiological and rule-based prediction models are still limited by the influence of various external factors and high inter and intra-subject variability on glucose dynamics^18^.

The widespread use of wearable devices and smartphone apps yields a substantial amount of granular data and has boosted machine learning-based algorithms in the literature^19^. Previous work has explored several classic machine learning approaches for the prediction of glucose levels or glycemic events^20–24^ using prediction horizons between 15 and 60 min. In a recent study, non-invasive wearable measurements combined with food logs were employed as digital biomarkers to estimate interstitial glucose using a machine learning method^25^. As indicated by a recent review^26^, deep learning technologies have attracted increasing attention in the field of diabetes, such as diabetic retinopathy^27,28^, neuropathy^29^, and glycemic control^30,31^. Empowered by various deep neural networks, deep learning has also achieved the state of the art in glucose prediction^26,32–38^ and has been applied to detect hypoglycemia using non-invasive vital signs, e.g., electrocardiograms (ECG)^39^.

**Fig. 1:**
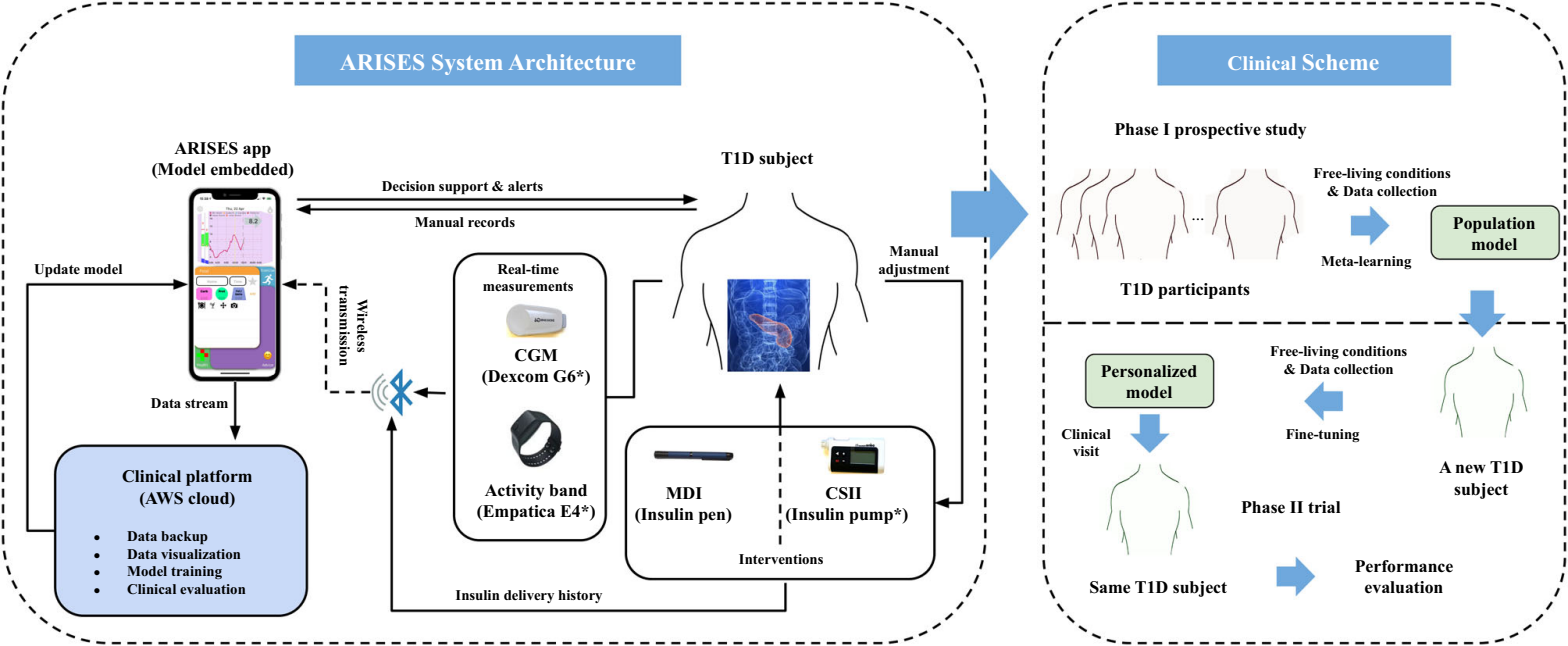
System architecture and clinical scheme of ARISES. A T1D subject is equipped with CGM and the wristband to measure glucose levels and vital signs, both of which communicate with the ARISES app via Bluetooth connectivity and provide input data for the deep learning models. The wearable devices in the system are marked by *. The data collected in phase I are used to train a population model with meta-learning, which is then fine-tuned in phase II to develop personalized models.

In this work, we introduce ARISES (Adaptive, Real-time, and Intelligent System to Enhance Self-care), a smartphone-based platform, to facilitate decision support and enhance self-management for people with T1D. It is based on an innovative mobile app with an embedded deep learning model for real-time glucose prediction and hypo- and hyperglycaemia warnings, which integrates data from CGM (Dexcom G6, Dexcom Inc., San Diego, CA, US) and a clinically validated physiological data acquisition sensor (Empatica E4 wristband, Boston, MA, US). In particular, we develop the prediction algorithm with an architecture of the recurrent neural network (RNN), leveraging a number of recent advances in deep learning, including attention mechanisms^40^, evidential regression^41^, and model-agnostic meta-learning (MAML)^42^. Fig. 1 shows the overall system architecture. The app interacts with the wearable devices via Bluetooth connections and has a new graphical user interface (Supplementary Fig. 1), which is specifically designed according to the feedback of T1D users, aiming to reduce cognitive burden and facilitate the visualization of information. The app allows users to record various daily activities, including meal composition, insulin injection, exercise, and health conditions, view the glucose trajectories, historical daily logs and predictions with the metaphor underlying the bifocal display^43^, and receive warnings of potential adverse glycemic events.

## Results

### Participant characteristics

Table 1 presents the demographic and clinical characteristics of the 12 T1D participants in the phase I prospective study. We collected a median (IQR) of 1113.5 (1059.0–1184.0) and 832.5 (733.0–953.0) hours of glucose data and sensor wristband data, respectively, and received a total of 5767 daily entries with a median (IQR) of 396 (237–732.3) interactions (Supplementary Table 1 and 2), including carbohydrates, protein, fat, insulin bolus, exercise, alcohol, stress, and illness, where the carbohydrate entries account for the largest portion.

**Table 1:** Demographic characteristics and clinical characteristics of the 12 T1D participants in the phase I clinical study.

Demographic characteristics	Median (IQR)
Age (years)	40.0 (30.0–49.0)
Gender (male/female)	6/6 (50.0% male)
Insulin regimen (CSII/MDI)^1^	6/6 (50.0% CSII)
HbA1c (mmol/mol)	50.4 (41.5–57.5)
Glucose data length (hours)	1113.5 (1059.0–1184.0)
Sensor wristband data length (hours)	832.5 (733.0–953.0)
Clinical characteristics	Mean ± SD
Time below range (<54 mg/dL) (%)	0.4 ± 0.3
Time below range (<70 mg/dL) (%)	2.9 ± 1.9
Time in range ([70, 180] mg/dL) (%)	63.4 ± 15.8
Time above range (>180 mg/dL) (%)	33.7 ± 16.9
Low blood glucose index	0.8 ± 0.5
High blood glucose index	7.6 ± 4.2
Average daily risk range	40.4 ± 10.5
Inter-day coefficient of variation (%)	35.2 ± 4.5
Intra-day coefficient of variation (%)	30.9 ± 4.8
Mean glucose level (mg/dL)	161.2 ± 25.9
Median glucose level (mg/dL)	154.8 ± 26.8

^1^*MDI* multiple daily injection, *CSII* continuous subcutaneous insulin infusion.

### Independent predictors using non-invasive physiological data

**Fig. 2:**
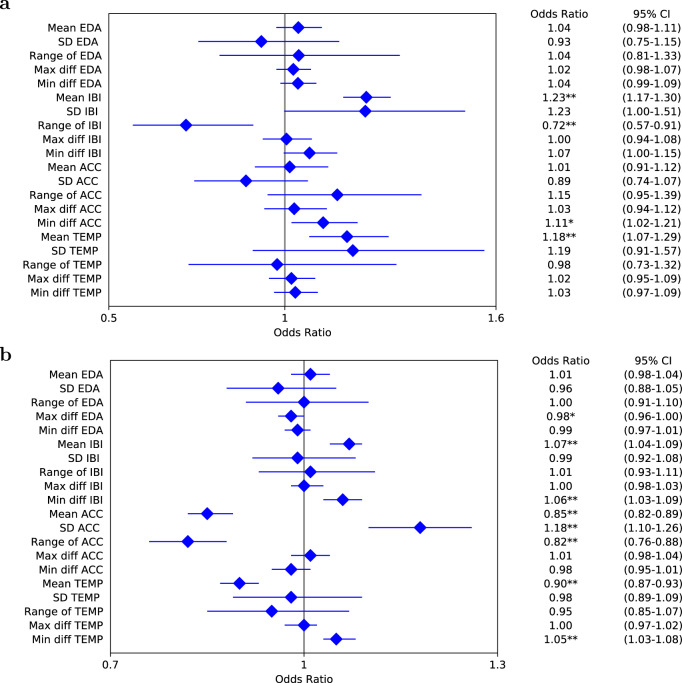
Forest plots of mixed effects logistic regression showing the association between non-invasive physiological measurements and adverse glycemic events. **a** Analysis for hypoglycemia. **b** Analysis for hyperglycemia. The measurements include electrodermal activity (EDA), inter-beat intervals (IBIs), acceleration (ACC), and skin temperature (TEMP). The horizontal error bars represent 95% confidence intervals (CIs). The regression coefficients were computed for mean values, standard deviation (SD), range, and maximum and minimum differential (diff) values over a one-hour retrospective window. The differential values refer to difference between adjacent measurements. The significance of a predictor is indicated as *p < 0.05, **p < 0.01.

The association of the non-invasive physiological measurements with adverse glycemic events over a 60-minute prediction horizon by mixed effects logistic regression is shown in Fig. 2. Hypoglycemia is negatively associated with a larger range of inter-beat intervals (IBIs) (odds ratio (OR): 0.72, 95% confidence interval (CI): 0.57–0.91; *p* < 0.01), while higher mean IBIs and mean skin temperature increases the OR of hypoglycemia (OR: 1.23, 95% CI: 1.17–1.30; *p* < 0.01; and OR: 1.18, 95% CI: 1.07–1.29; *p* < 0.01, respectively). Similarly, we observe that, besides the IBIs and skin temperature, variables derived from electrodermal activity (EDA) and acceleration are also significant predictive factors for hyperglycemia prediction. Considering all the physiological signals are significantly associated with adverse glycemic events, we, therefore, combined these non-invasive measurements with CGM and daily entries to extract a total of 20 real-time features (Supplementary Table 3), which were used in feature selection for the deep learning-based prediction model.

### Glucose level prediction

Table 2 presents the performance of the personalized ARISES model with 15, 30, 45, 60-minute prediction horizons. The proposed model outperformed all the considered baseline methods in terms of root mean square error (RMSE), glucose-specific RMSE (gRMSE), mean absolute error (MAE), mean absolute percent error (MAPE), and the time lag. The results of the baseline methods are presented in Supplementary Table 2. The ARISES obtained significant improvement in RMSE, gRMSE, MAE, and MAPE, when compared with the best performance of the baseline methods (convolutional recurrent neural networks (CRNNs)^34^; *p* < 0.01). When only one day of data was used for fine-tuning, the MAML approach obtained the average RMSE of 39.37 ± 7.14 for the 60-minute prediction horizon, which is much smaller than the RMSE obtained by a baseline method of transfer learning^36^ (RMSE: 43.07 ± 8.41; *p* < 0.05).

**Table 2:** Results of glucose level prediction (Mean ± SD) evaluated on 12 clinical T1D subjects.

Prediction horizons	15 min	30 min	45 min	60 min	60 min (Baseline^34^)
RMSE (mg/dL)	10.15 ± 1.67	20.92 ± 3.55	28.99 ± 4.41	35.28 ± 5.77	37.18 ± 6.09**
gRMSE (mg/dL)	12.14 ± 2.06	26.07 ± 4.47	37.20 ± 5.97	46.26 ± 7.73	49.04 ± 8.50**
MAE (mg/dL)	7.21 ± 1.09	15.06 ± 2.36	21.15 ± 3.15	26.11 ± 4.36	27.77 ± 4.89**
MAPE (%)	5.07 ± 0.97	10.62 ± 2.03	14.94 ± 2.77	18.53 ± 3.78	19.23 ± 4.07**
Time lag (min)	1.39 ± 1.06	7.37 ± 5.18	14.00 ± 7.24	17.63 ± 11.39	21.57 ± 11.41

Root mean square error (RMSE), glucose-specific RMSE (gRMSE), mean absolute error (MAE), mean absolute percent error (MAPE), and the time lag are employed as metrics. The best performance of the baseline methods (CRNN^34^) is presented.

The significance is indicated as **p < 0.01.

**Fig. 3:**
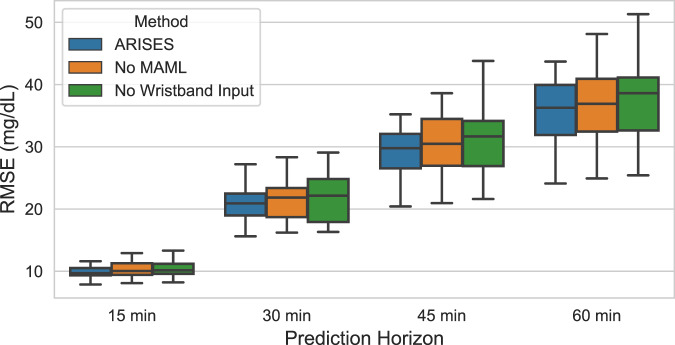
Ablation analysis on the prediction performance of glucose levels. The model achieved smaller average RMSE for the 12 T1D subjects when using MAML and wristband input data. The improvement is most significant for the 60-minute prediction horizon. The lower and upper hinges of boxplots show the first quarter (Q1) and the third quartile (Q3), respectively. The central lines indicate the median, while the whiskers extend to 1.5 IQR.

In addition, Fig. 3 shows the results of ablation analysis, where we removed certain components from the model and evaluated their impact on the prediction performance. In particular, the use of MAML and wristband input respectively reduced the average RMSE by 1.41 and 2.25 mg/dL (*p* < 0.05) for the 60-minute prediction horizon.

### Hypoglycemia and hyperglycemia prediction

**Fig. 4:**
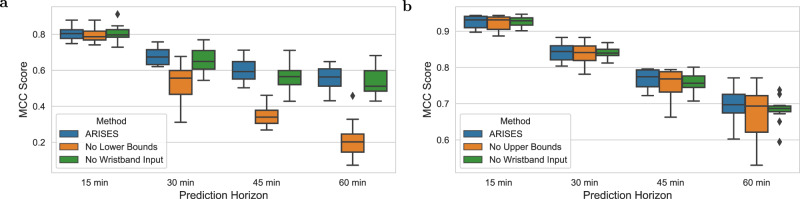
Ablation analysis on the prediction of adverse glycemic events, evaluated on the 12 T1D subjects. **a** MCC scores for hypoglycemia prediction. **b** MCC scores for hyperglycemia prediction. The lower bounds significantly improved hypoglycemia prediction, while the use of wristband data enhanced MCC scores for all the prediction horizons. The lower and upper hinges of boxplots show the Q1 and the Q3, respectively. The central lines indicate the median, while the whiskers extend to 1.5 IQR.

Tables 3 and 4 respectively show the results of hypoglycemia and hyperglycemia prediction using the lower and upper bounds derived from evidential deep learning. We observe that the proposed ARISES model achieved the accuracy of 88.58% with the sensitivity of 70.30% and the accuracy of 87.20% with the sensitivity of 86.62% for hypoglycemia and hyperglycemia prediction over the 60-minute prediction horizon, respectively. For the considered baseline methods (Supplementary Tables 5 and 6), we used the predicted glucose levels, i.e., single trajectories, to detect adverse glycemic events. Among these, the autoregressive moving average (ARMA)^44^ and the physiologically-based kinetic model (PKM)^45^ achieved the best baseline results for hypoglycemia and hyperglycemia prediction, respectively, which are reported in Tables 3 and 4 for comparison. It is worth noting that, compared with the ARMA, the ARISES model significantly increased sensitivity by 13.35% and reduced the mean deviation (MD) by 13.29 mg/dL for hypoglycemia prediction. Compared with the PKM for hyperglycemia prediction, the ARISES model significantly increased specificity and precision by 5.38% and 5.43%, respectively, while reducing the MD by 13.80 mg/dL. As shown in Fig. 4, we observe that the use of lower bounds and wristband input data enhanced the average Matthews correlation coefficient (MCC) scores by 0.34 (*p* < 0.01) for hypoglycemia prediction with the 60-minute prediction horizon.

**Table 3:** Results of hypoglycemia prediction (Mean ± SD) evaluated on 12 clinical T1D subject.

Prediction horizons	15 min	30 min	45 min	60 min	60 min (Baseline^44^)
Accuracy (%)	98.03 ± 1.03	94.96 ± 2.92	91.97 ± 4.22	88.58 ± 6.53	91.89 ± 5.23
Sensitivity (%)	84.15 ± 4.20	76.08 ± 5.88	72.07 ± 4.45	70.30 ± 12.84	56.95 ± 19.24**
Specificity (%)	98.72 ± 0.75	96.42 ± 2.48	93.99 ± 4.08	90.09 ± 8.21	94.87 ± 3.67
Precision (%)	78.91 ± 4.31	65.65 ± 5.31	58.23 ± 10.21	56.20 ± 10.43	55.28 ± 17.38
MCC score	0.80 ± 0.04	0.68 ± 0.05	0.60 ± 0.06	0.56 ± 0.07	0.51 ± 0.12
MD (mg/dL)	10.28 ± 3.80	19.18 ± 7.00	26.30 ± 9.28	28.63 ± 11.00	41.92 ± 14.60**

*MCC* Matthews correlation coefficient, *MD* mean deviation from true glucose levels for missed predicted hypoglycemic events. The best performance of the baseline methods (ARMA^44^) is presented.

The significance is indicated as **p < 0.01.

**Table 4:** Results of hyperglycemia prediction (Mean ± SD) evaluated on 12 clinical T1D subjects.

Prediction horizons	15 min	30 min	45 min	60 min	60 min (Baseline^45^)
Accuracy (%)	96.75 ± 0.99	93.22 ± 1.24	90.06 ± 1.05	87.20 ± 1.95	85.54 ± 3.13
Sensitivity (%)	95.32 ± 2.16	91.25 ± 4.75	88.48 ± 7.87	86.62 ± 7.81	91.58 ± 3.52
Specificity (%)	96.95 ± 1.14	92.62 ± 2.89	87.61 ± 5.10	82.59 ± 7.96	77.21 ± 4.74*
Precision (%)	94.62 ± 2.37	90.51 ± 3.20	87.43 ± 4.76	85.11 ± 5.83	79.68 ± 12.49*
MCC score	0.92 ± 0.02	0.84 ± 0.02	0.77 ± 0.03	0.70 ± 0.05	0.68 ± 0.06
MD (mg/dL)	13.00 ± 2.30	28.69 ± 5.16	40.05 ± 8.04	47.62 ± 10.33	61.42 ± 16.26**

*MCC* Matthews correlation coefficient. *MD* mean deviation from true glucose levels for missed predicted hyperglycemic events. The best performance of the baseline methods (PKM^45^) is presented.

The significance is indicated as *p < 0.05, **p < 0.01.

## Discussion

This study proposes a deep learning algorithm embedded in a smartphone-based platform to predict glucose levels and hypo- and hyperglycemia, with the input of CGM, daily entries, and real-time measurements from the physiological sensor wristband. Notably, the integration of the wristband data has improved the results of both glucose level prediction and hypo- and hyperglycemia detection.

**Fig. 5:**
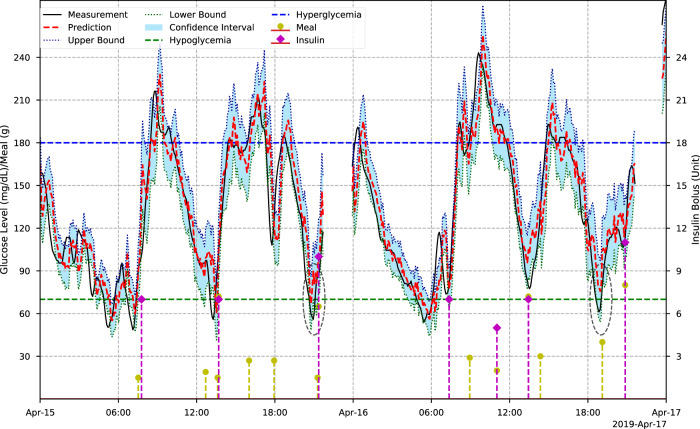
Two-day period CGM and prediction trajectories of a T1D adult over a 30-minute prediction horizon. The ellipses indicate the hypoglycemic events that are missed by prediction values but detected by the lower bounds.

Figure 5 depicts the predicted trajectories and CGM measurements of a participant over a two-day period. We present the 7-day trajectories of four selected participants in Supplementary Fig. 2 We observe that the daily activities, including meal intake and insulin bolus delivery, have a significant impact on the glucose levels. The glycemic homeostasis is affected by these external factors and internal changes in the T1D subject. Thus, the accuracy degrades as the prediction horizon increases in Tables 2, 3 and 4.

As a safety-critical system, the reliability of predictions is essential, especially when glucose levels are approaching the threshold of hypoglycemia. In clinical settings, the occurrence of hypoglycemia is more dangerous than that of hyperglycemia, which may lead to life-threatening complications^46^. To this end, we used an evidential deep learning approach^41^ to train the models and map model uncertainty. Most previous studies used mean square error as the loss function and computed a single prediction value to indicate whether there will be risk of hypo- and hyperglycemia^20,21,26,33,35^. However, hypo- and hyperglycemic events may fail to be detected when the confidence of a prediction is low. In the experiments, we noted that hypoglycemic events with short duration were likely to be missed when single trajectory values are used in detection (Fig. 5). Therefore, we use lower and upper bounds derived to determine adverse glycemic events and assist decision support in T1D self-management with the ARISES app (Supplementary Fig. 1). Displaying these informative bounds on the app is a preferable feature according to the requirements of the phase I participants. As highlighted by the eclipses in Fig. 5, the use of lower bounds successfully identified two hypoglycemic events that are likely to be missed using single prediction values.

The proposed ARISES model has achieved superior performance and outperformed six considered baseline methods (Supplementary Tables 2, 5 and 6). It is observed that the machine learning and deep learning baseline models obtained better RMSE performance for glucose level prediction, but smaller MCC scores for hypo- and hyperglycemia prediction, when compared with the physiological and statistical baseline methods. One possible explanation is that the machine learning and deep learning baseline models were optimized in a supervised learning process with the targets of actual CGM measurements, but the prediction of adverse glycemic events was not considered. In this regard, the introduced lower and upper bounds in the ARISES model enabled a good balance between glucose level prediction and hypo- and hyperglycemia detection.

We compared the MCC scores using these bounds against the results of single curve prediction in Fig. 4, where the classification based on the bounds exhibited better performance. We noticed that hypoglycemia is a minority class in the dataset, which accounts for 2.91 ± 1.93% of total glucose measurements (Table 1). In general, the classifier is less sensitive to detecting a minority class. Nevertheless, in this work, the sensitivity can be further enhanced by reducing the thresholds of lower bounds at a cost of potential alarm fatigue. This trade-off can be decided by clinicians on an individual case basis.

We used the MAML approach to train population models and personalized models, which outperformed the transfer learning approach with a small amount of available data. This fast adaption feature of the MAML approach can mitigate the cold-start issues when we provide the software to new T1D users with limited personal data. It is a common scenario in actual clinical settings since data collection is expensive and time-consuming. Moreover, the MAML also improved the final average RMSE results in the ablation analysis (Fig. 3).

The chronological partition of training, validation and testing set in this work was carefully selected. Random cross-validation can be found in previous work, which trained and validated machine learning models on the same dataset^24,39^. However, during the experiments, we noticed that there were temporal dependencies between the data points from nearby locations, especially in adjacent ones. The features were derived with the small resolution of CGM, so the difference between consecutive time steps is sometimes negligible. In this regard, the use of random or stratified splitting methods would introduce underlying temporal correlation into training and testing sets, which could result in serious overestimation of model accuracy^47^.

The ARISES app (Supplementary Fig. 1) is based on the iOS operating system and integrates with Dexcom CGM (G5 or G6) and Empatica E4 wristband. The source code of the app is not publicly available. We analyzed the performance of the app on an iPhone XS Max over 50 runs. The whole app has an initial storage size of 39.9 MB and consumed an average of 50.5 MB and 39.3 MB memory while running in foreground and background, respectively. The trained deep learning models were converted to mobile compatible format via TensorFlow Lite, which has a storage size of 1.2 MB. When the app received a new CGM measurement, it took 5.7 ms and 1.8 MB memory to compute real-time glucose prediction through model inference on the edge, which require one-hour historical data of CGM measurements, sensor wristband measurements, and daily entries (if any). Model fine-tuning is performed by Amazon S3 buckets and SageMaker in the Amazon Web Services (AWS) cloud (Fig. 1) and requires at least one-week historical data.

Our results suggest that measurements obtained from wearable physiological wristband data sensors could be integrated alongside CGM data to improve the prediction of glucose levels and adverse glycemic events. Interestingly, the IBI measured by the sensor wristband is the only predictor that has significant effect on both hypoglycemia and hyperglycemia prediction (Fig. 2), which was also selected as the input of the deep learning model with the best validation performance. It indicates IBI or other heart rate variability (HRV) could be useful biomarkers in T1D decision support, which accords with the findings of previous studies^48–50^. However, the sensors in Empatica E4 are quite sensitive to motion artifacts, so it is difficult to obtain accurate measurements with too many hand movements^51^. In future work, an algorithm to detect exercise and reduce measurement error for the wristband will be developed. Meanwhile, data extracted from manually recorded daily events have the potential to be used for the analysis of the drivers and patterns of the changes in plasma or interstitial glucose concentrations. During feature pre-processing, we calculated insulin on board and the carbohydrate on board with fixed duration (i.e., time of decay) and constant absorption rate of carbohydrates, respectively. Nonlinear insulin on board and carbohydrate on board based on physiological models with personalized parameters., such as the variable appearance rate of glucose and plasma insulin estimation^52^, will be considered in the future, aiming to improve quality of input features and further enhance prediction accuracy. We collected the dietary data from the T1D participants under free-living conditions, so the dietary reporting is variable in quality but reflects the real-world use of carbohydrate counting and self-management. Although we manually checked the carbohydrate amount for each meal record to confirmed that there are no unrealistic values, such as negative or larger than 500 g, it would be interesting to investigate how the accuracy of dietary reporting affects prediction performance, which could be done by analyzing the results obtained from datasets collected in inpatient trials with standardized meals. It is noted that the percentages of time spent below range (Table 1) are small, and there is a modest carbohydrate intake of 160 (102–220) grams per day (Supplementary Table 2). Although these values are not unusual for people living with T1D, especially for those who use CGM to visualize post-prandial glucose peaks, it is a potential limitation in the development of the algorithm to predict hypoglycemic events. Future work will include validating the proposed system on a T1D cohort with greater variance in carbohydrate intake and glycemic variability. Currently, there are no publicly available T1D datasets containing all the data fields that we need in the ARISES model, but it is important to further test the generalization of the proposed algorithm using an independent validation dataset with a larger cohort size. In this case, we also recommend to analyze covariates in the T1D population, such as age and insulin delivery mode. In addition, there is a lack of system testing of the whole ARISES in real-world settings. It might be challenging to simultaneously administer the multiple wearable devices, smartphone app, and cloud services with reliable wireless connectivity. A deep learning model with only CGM input and daily entries needs to be implemented as a sub-optimal solution when the wristband data is not available, e.g., when the wristband is taken off for battery charging.

## Methods

### Phase I prospective study

This was a six-week longitudinal prospective study (NCT ID: NCT03643692) using a clinically validated real-time physiological data acquisition sensor (Empatica E4) and CGM (Dexcom G6) to identify correlations between measurable physiological parameters and glycemia. Under free-living conditions, twelve adults (18 years old and older) with a median age (IQR) of 40 years (30–50) were equally stratified by gender and mode of insulin delivery (MDI and CSII). Participants were recruited from the Imperial College Healthcare NHS trust T1D outpatient clinics, registered research databases, and interested participants who contact us. Throughout the duration of the study, participants wore the Empatica E4 and Dexcom G6 devices with alarm thresholds of glucose levels set at <4 mmol/L and >11 mmol/L. Participants were asked to log daily events such as, insulin doses in units, meal macronutrient composition in grams, alcohol intake in units, stress, illness, and exercise in the mySugr smartphone app, which are used to develop the input features of glucose prediction models. The study was conducted under a trial protocol (18/LO/1096) approved by London - Fulham Research Ethics Committee, and each participant was informed and signed consent.

### Analysis of sensor wristband data

Different from most of the previous studies using CGM and daily manual logs^20–24,26^, an objective of this work is to better understand the effect of the non-invasive physiological data on the prediction of glycemic events. Using the package lme4 in R, a mixed effects logistic regression was applied calculate the logarithm of ORs to interpret the relationship between physiological measurements and the binary outcome of adverse glycemic events (i.e., hypoglycemia (<70 mg/dL) or hyperglycemia (> 180 mg/dL) in Fig. 2^53^. The measured physiological variables applied to the regression analysis include the mean values, standard deviation, range, and maximum and minimum differential values of EDA, IBI, acceleration, and skin temperature signals.

### Multi-modal feature engineering

As a clinically validated, commercially available, and non-invasive device, the Empatica E4 wristband uses a photoplethysmography sensor to measure blood volume pulse (BVP), two electrodes to obtain EDA, a pair of accelerometers and a gyroscope to detect the level of physical activity, and a peripheral temperature sensor to monitor skin temperature. In previous clinical studies, BVP and ECG signals are the primary sources to identify IBIs for HRV analysis^54^. In particular, we applied a band-pass Butterworth filter to remove noise in BVP signals and employed a slope sum function^55^ to detect the local maxima. Then we used a sliding window with decision rules^55^ to search peaks, as the systoles in cardiac cycles. The IBIs were computed by the difference of consecutive peaks.

We extracted short-term HRV features with a 5-minute window to indicate early HRV changes^48^ in temporal and frequency domains^56^. To obtain skin conductance levels (SCLs) and skin conductance responses (SCRs), we continuously decomposed EDA data into tonic and phasic components via a high-pass filter^57^. There are two open-source software tools involved in EDA and BVP processing^58,59^. Together with physical activity levels and skin temperature, the outcomes of these features in the past five minutes were averaged and aligned with the time steps of CGM readings. HRV is an established indicator that reflects cardiac autonomic activities, while EDA is related to the status of the nervous system. These biomarkers have been used in previous studies to predict and detect hypoglycemia for T1D^48,50,60^.

The daily entries were converted to insulin on board and carbohydrate on board via physiological models. We assumed insulin bolus lasts for four hours in the human body with different slopes, as a common setting used by many commercialized pumps^24,61^. Similarly, the carbohydrate was assumed to be absorbed at a rate of 2.5 g/min 15 min after the time of meal ingestion^24^.

### Feature pre-processing and selection

We obtained a total of 20 features from the pre-processed multi-modal data (Supplementary Table 3). There are some inevitable errors in the sensor data, e.g., compression artifacts, signal loss, and sensor calibration. To this end, we performed feature selection in the following steps. First, we analyzed the missing fraction of CGM and wristband measurements to identify the quality of features. The median value of the missing percentages of CGM and wristband data are 3.02% and 23.05%, respectively, which are reasonable since the wristband needs to be charged for around 4–5 hours every day. We linearly interpolated the gaps that occurred in the middle of input sequences and extrapolated the gaps at the tail to guarantee that future information is not involved in current predictions. Then, min-max normalization was adopted to scale the selected features to [0, 1]. Finally, we performed collinearity analysis, considering correlated bias is prone to degrade the stability and interpretability of machine learning models^62^. We noted that features derived from the same measurement exhibited strong a correlation with each other. Hence, each time we retained one feature in IBI or EDA feature group (Supplementary Table 3) and selected the best combination according to the error scores that summed up RMSE results for the four prediction horizons in model validation.

### Model training, validation, and testing

Considering the personalized models are provided to the T1D subjects at a midterm clinical visit (Fig. 1), we divided the data of each subject into a training set and a testing set that include the first 50% data and the remaining 50% data, respectively. The last 20% data of each training set were used as a hold-out personalized validation set. To simulate a clinical scheme with two phases (Fig. 1), we employed a population set containing the training sets of 11 subjects and a personalized set with the data of the remaining subject, assuming it is a new subject (e.g., a participant in phase II). Data of each subject in the population set were used to optimize the population model. The population models were validated with leave-one-subject-out cross validation. Then we used the training data of the personalized set to fine-tune the population model to develop a personalized model, and used the testing data of the personalized set for evaluation. The Hyperband algorithm^63^ of Keras tuner was used to select the best hyperparameters of the deep learning models (Supplementary Table 7). Besides, we used early stopping to mitigate overfitting and improve generalization.

### Developing population and personalized models

With the population and personalized data sets, we applied a well-established MAML framework to develop population models^42^. Each subject is regarded as a learning task in the inner loop of MAML. Then, the learned parameters for each task guided the population model to achieve meta-optimization via stochastic gradient descent in the outer loop. The first-order approximation was performed to accelerate the training process^64^. In the personalization phase, we fine-tuned the meta-model with a small learning rate^65^.

We performed an experiment to compare the MAML population model with the pre-trained model by classic transfer learning^36^. For each subject, the data collected on the fist day of the trial, i.e., the first 288 data samples (5-minute CGM resolution) in a personalized training set, were used to fine-tune both models. Then, we evaluated the performance of the fine-tuned models using the testing data of the personalized set.

### Attention-based RNN architecture

**Fig. 6:**
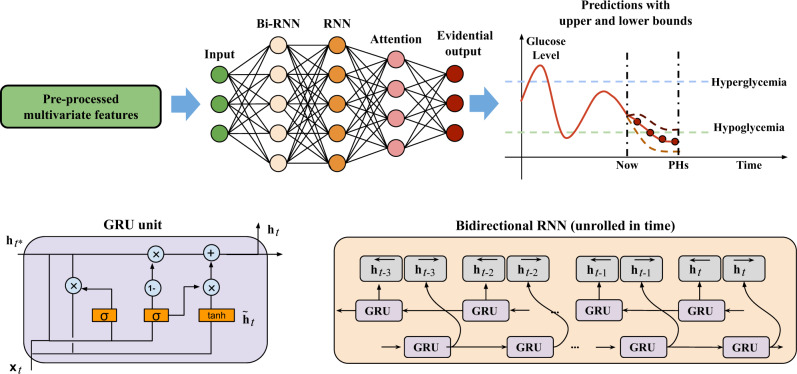
Architecture of the deep learning model. A stack of bidirectional RNN (Bi-RNN) and RNN with GRU cells is used to extract hidden representations from the input multivariate time series. With the weighted state vector by attention layer, the evidential output computes glucose predictions along with model uncertainty.

The recurrent structure is well-suited to learn short and long-term temporal dependencies in sequence processing. Thus, RNN-based models are emerging in the literature of diabetes management and have been shown to exhibit superior performance in glucose prediction^26,33,36,66^. However, the vanilla RNNs face the challenge of gradient exploding and vanishing, which largely limits the learning performance on long-term temporal dependencies. Fortunately, long short-term memory (LSTM)^67^ and gated recurrent units (GRUs)^68^ were proposed to solve this problem. The GRU uses reset and update gate functions with less parameters than LSTM (Fig. 6)^36^.

After pre-processing the features, we developed an attention-based RNN with GRUs for glucose prediction and hypo- and hyperglycemia detection. The multivariate input data for the RNN model were selected according to validation performance, which include CGM, carbohydrate amount, insulin bolus, time index, IBIs, and SCRs.

At each time step *t* with a CGM measurement *G*_*t*_, the target of the algorithm is to predict a glucose level at *t* + *w*, where *w* is calculated as the prediction horizon divided by the CGM resolution. Here, we define the normalization function as *m* and the prediction targets as *y*_*t*_ = *m*(*G*_*t*+*w*_ − *G*_*t*_), using glucose change to minimize the bias^35,36^. The model input consists of a multidimensional vector ${{{{\bf{X}}}}}_{t}={[{{{{\bf{x}}}}}_{t},{{{{\bf{x}}}}}_{t-1},\ldots ,{{{{\bf{x}}}}}_{t+1-l}]}^{{\mathsf{T}}}\in {{\mathbb{R}}}^{l\times c}$, where *l* is the length of the input sequence; *c* is the feature dimension.

Leveraging the previous and future information in a sequence, we modeled a bidirectional structure to simultaneously compute two states in forward direction (${\overrightarrow{{\mathop{{\bf{h}}}}}}_{t}$) and backward direction ($\overleftarrow{{\bf{h}}}_t$) and merged them into an output vector ${{{\mathbf{h}}}}_t^b = \left[ {\overleftarrow {{{{\mathbf{h}}}}_t} ;\,\overrightarrow {{{{\mathbf{h}}}}_t} } \right]$^32,69^. Fig. 6 correspondingly illustrates the unfolded block diagrams. The output of the bidirectional RNN is sent to another GRU-based RNN layer to obtain high-level hidden representations, of which the cell output is denoted as **h**_*t*_.

We employed the attention mechanism, as one of the latest advances in deep learning, to extract temporal dependencies regardless of distance. Introducing attention in deep neural networks has shown success in a variety of tasks, especially in natural language processing^70^. Instead of only using the output of the final state, the attention mechanism assigns attention weights to the hidden state **h**_*t*_ on each time step and then combines them to compute the final representation vector. In particular, we modified the general form of Luong’s multiplicative attention^40^ and implemented the many-to-one attention weight *a*_*t*_ at time step *t* as:

1 $${a}_{t}=\frac{\exp ({{{{\bf{h}}}}}_{T}^{\top }{{{{\bf{W}}}}}_{a}{{{{\bf{h}}}}}_{t})}{\mathop{\sum}\nolimits_{t^{\prime} }\exp ({{{{\bf{h}}}}}_{T}^{\top }{{{{\bf{W}}}}}_{a}{{{{\bf{h}}}}}_{t^{\prime} })}$$

where ${{{{\bf{h}}}}}_{T}^{\top }$ is the final cell state. The attention output vector **v** is computed with hidden weights **W**_*v*_ and $\tanh$ activation, which is defined as follows

2 $${{{\bf{v}}}}=\tanh ({{{{\bf{W}}}}}_{v}[\mathop{\sum}\limits_{t}{a}_{t}{{{{\bf{h}}}}}_{t};{{{{\bf{h}}}}}_{T}]),$$

### Lower and upper bounds of predictions

To determine the reliability and confidence of the predictions, model uncertainty is estimated by a higher-order evidential distribution^41^. Assuming the predictions are drawn from a Gaussian distribution with unknown mean *μ* and variance *σ*^2^, i.e., $\mu \sim {{{\mathcal{N}}}}(\gamma ,{\sigma }^{2}/\lambda )$, *σ*^2^ ~ Γ^−1^(*α*, *β*), we fed the attention output vector into an evidential layer to map the normal-inverse-gamma distribution as (*μ*, *σ*^2^) ~ N − Γ^−1^(*γ*, *λ*, *α*, *β*). In this case, the final model output comprises four parameters (*γ*_*t*_, *λ*_*t*_, *α*_*t*_, *β*_*t*_), which are computed by a dense layer with four neurons, where ${\hat{y}}_{t}={\gamma }_{t}$. The output of the evidential layer (evid) and epistemic uncertainty (i.e., model uncertainty) *u*_*t*_ are defined as

3 $${\hat{y}}_{t},{\lambda }_{t},{\alpha }_{t},{\beta }_{t}={{{\rm{evid}}}}({{{\bf{v}}}}),\quad {u}_{t}=\sqrt{\frac{{\beta }_{t}}{{\lambda }_{t}({\alpha }_{t}-1)}}.$$

Thus, the corresponding glucose prediction ${\hat{G}}_{t+w}$, lower bound ${B}_{t+w}^{l}$, and upper bound ${B}_{t+w}^{u}$ can be denoted as

4 $$\begin{array}{ll}{\hat{G}}_{t+w}={G}_{t}+{m}^{-1}({\hat{y}}_{t}),\\ {B}_{t+w}^{l}={\hat{G}}_{t+w}-{k}^{l}{m}^{-1}({u}_{t}),\\ {B}_{t+w}^{u}={\hat{G}}_{t+w}+{k}^{u}{m}^{-1}({u}_{t}),\end{array}$$

where *m*^−1^ is the inverse function of the normalization; *k*^*l*^ and *k*^*u*^ are the thresholds of the uncertainty. Clinicians are allowed to adjust the thresholds to obtain specific clinical efficacy. For instance, increasing the value of *k* can enhance the sensitivity of the classifier to avoid missing the warnings of adverse glycemic events. During the model training, the negative log-likelihood loss function to optimize the parameters with maximum likelihood estimation can be solved by a Student-t distribution according to Bayesian probability theory^41^.

### Performance evaluation

The glucose predictions were estimated by the mean values of the evidential distribution of the model output. The regression performance was evaluated by the RMSE, gRMSE, MAE, MAPE, and the time lag. In particular, gRMSE penalizes the prediction errors that could lead to harmful events, such as overestimation in hypoglycemia and underestimation in hyperglycemia, to demonstrate clinical impact ^71^, which is defined as follows:

5 $$\begin{array}{ll}{{{\rm{gRMSE}}}}\qquad\qquad=\sqrt{\frac{1}{N}\mathop{\sum }\limits_{t=1}^{N}P({G}_{t+w,}{\hat{G}}_{t+w}){({G}_{t+w}-{\hat{G}}_{t+w})}^{2}},\\ P\left.({G}_{t+w},{\hat{G}}_{t+w})\right)=1+{\alpha }_{L}{\bar{\sigma }}_{{G}_{t+w}\le {T}_{L},{\beta }_{L}}({G}_{t+w}){\sigma }_{{\hat{G}}_{t+w}\ge {G}_{t+w},{\gamma }_{L}}({\hat{G}}_{t+w},{G}_{t+w})\\ \qquad\qquad\qquad\qquad+\,{\alpha }_{H}{\sigma }_{{G}_{t+w}\ge {T}_{H},{\beta }_{H}}({G}_{t+w}){\bar{\sigma }}_{{\hat{G}}_{t+w}\le {G}_{t+w},{\gamma }_{H}}({\hat{G}}_{t+w},{G}_{t+w}),\end{array}$$

where $P({G}_{t+w,}{\hat{G}}_{t+w})\ge 1$, and values of *α*_*L*_, *β*_*L*_, *γ*_*L*_, *T*_*L*_, *α*_*H*_, *β*_*H*_, *γ*_*H*_, *T*_*H*_ equal to 1.5, 30, 10, 85, 1, 100, 20, 155, respectively. The time lag is derived by the cross-correlation of predicted glucose levels and actual CGM measurements^34,35^, which denotes the time-shift between two time series. A smaller time lag indicates a faster response of the prediction method to the changes in CGM trends and thus better prediction performance.

The thresholds of lower and upper bounds were selected in model validation according to MCC scores, which are respectively used to detect hypoglycemia and hyperglycemia. In particular, a hypoglycemic or hyperglycemic event is defined as three consecutive CGM measurements (i.e., at least 15 min) below 70 mg/dL or above 180 mg/dL, as recommended by previous studies^72^. A true positive means that an adverse glycemic event is correctly identified, while a false negative indicates a missed prediction. We evaluated the classification performance of hypo- and hyperglycemia prediction using a set of standard metrics, including accuracy, sensitivity, specificity, precision, and MCC^22–24^. Good MCC scores can be obtained only if the classifier performs well in all confusion matrix categories, which is a more reliable and informative score than accuracy and the F1 score in binary classification^73^. In addition, we introduced MD scores calculated by the MAE for the glucose sequences in missed predicted hypoglycemic or hyperglycemic events.

We used the results for the 60-minute prediction horizon as the primary outcomes, since predicting glucose over such a long prediction horizon is challenging. The converted TensorFlow Lite models were evaluated to simulate on-device inference in the ARISES app. To compare the proposed model with existing approaches, we employed a set of classic machine learning and deep learning baseline methods (Supplementary Tables 2, 5 and 6), including support vector regression (SVR) with the RBF kernel^21^, artificial neural networks (ANNs) with three fully-connected layers^20^, bidirectional long short-term memory (Bi-LSTM)^32^, and CRNNs^34^. Besides, we also used a statistical model, the ARMA with exogenous inputs^44^, and a physiological model, the PKM, which is based on the composite minimal model of plasma glucose and insulin kinetics with personalized insulin sensitivity, time to maximum glucose rate of appearance, and time to maximum insulin absorption^45,74^. The PKM has been validated on both the in silico data from the UVA/Padova T1D simulator^75^ and real data from clinical trials^76^ in terms of glucose prediction^45^. The input features of baseline models were identical to those of the proposed model, except that the PKM only used the information of CGM measurements, carbohydrate intake and insulin bolus. To calculate the statistical significance with respect to the considered baseline results, we performed paired *t* tests after evaluating the normality by Shapiro–Wilk tests.

### Reporting summary

Further information on research design is available in the Nature Research Reporting Summary linked to this article.

## Supplementary information

Reporting Summary

Supplementary Information

Clinical Trial Protocol

## Data Availability

The dataset used in this study is not publicly available due to the proprietary nature of the data and privacy concerns. Interested researchers should contact the corresponding authors to inquire about the access.

## Code availability

The free and open-source programming languages R 3.6.3 and Python 3.8.5 with the deep learning framework TensorFlow 2.2.0 were used in this study. The source code of the deep learning model and smartphone app is available upon reasonable request from the corresponding authors.

## References

[1] Saeedi P (2019). Global and regional diabetes prevalence estimates for 2019 and projections for 2030 and 2045: Results from the International Diabetes Federation Diabetes Atlas. Diab. Res. Clin. Practice.

[2] Katsarou A (2017). Type 1 diabetes mellitus. Nat. Rev. Disease Primers.

[3] Yale J-F, Paty B, Senior PA (2018). Hypoglycemia. Can J Diabetes.

[4] Gregg EW, Sattar N, Ali MK (2016). The changing face of diabetes complications. Lancet Diabetes Endocrinol..

[5] Rodbard D (2016). Continuous glucose monitoring: a review of successes, challenges, and opportunities. Diabetes Technol. Therapeutics.

[6] Juvenile Diabetes Research Foundation Continuous Glucose Monitoring Study Group. Continuous glucose monitoring and intensive treatment of type 1 diabetes. *N. Engl. J. Med.* **359**, 1464–1476 (2008) . 10.1056/NEJMoa0805017 18779236

[7] Heinemann L (2018). Real-time continuous glucose monitoring in adults with type 1 diabetes and impaired hypoglycaemia awareness or severe hypoglycaemia treated with multiple daily insulin injections (HypoDE): a multicentre, randomised controlled trial. Lancet.

[8] Herrero P, Georgiou P, Oliver N, Johnston DG, Toumazou C (2012). A bio-inspired glucose controller based on pancreatic *β*-cell physiology. J. Diabetes Sci. Technol..

[9] Oliver N, Reddy M, Marriott C, Walker T, Heinemann L (2019). Open source automated insulin delivery: addressing the challenge. npj Digital Med..

[10] Kirwan M, Vandelanotte C, Fenning A, Duncan MJ (2013). Diabetes self-management smartphone application for adults with type 1 diabetes: randomized controlled trial. J. Med. Internet Res..

[11] Ryan EA (2017). Improved A1C levels in type 1 diabetes with smartphone app use. Can. J. Diabetes.

[12] Sevil M (2020). Determining physical activity characteristics from wristband data for use in automated insulin delivery systems. IEEE Sensors J..

[13] Ozaslan B, Patek SD, Breton MD (2020). Impact of daily physical activity as measured by commonly available wearables on mealtime glucose control in type 1 diabetes. Diabetes Technol. Ther..

[14] Wu Y (2017). Mobile app-based interventions to support diabetes self-management: a systematic review of randomized controlled trials to identify functions associated with glycemic efficacy. JMIR mHealth uHealth.

[15] Lithgow K, Edwards A, Rabi D (2017). Smartphone app use for diabetes management: evaluating patient perspectives. JMIR Diabetes.

[16] Mathieu C, Gillard P, Benhalima K (2017). Insulin analogues in type 1 diabetes mellitus: getting better all the time. Nat. Rev. Endocrinol..

[17] Battelino T, Nimri R, Dovc K, Phillip M, Bratina N (2017). Prevention of hypoglycemia with predictive low glucose insulin suspension in children with type 1 diabetes: a randomized controlled trial. Diabetes Care.

[18] Herrero P (2017). Enhancing automatic closed-loop glucose control in type 1 diabetes with an adaptive meal bolus calculator–in silico evaluation under intra-day variability. Comput. Methods Progr. Biomed..

[19] Woldaregay AZ (2019). Data-driven blood glucose pattern classification and anomalies detection: machine-learning applications in type 1 diabetes. J. Medical Internet Res..

[20] Pérez-Gandía C (2010). Artificial neural network algorithm for online glucose prediction from continuous glucose monitoring. Diabetes Technol. Ther..

[21] Georga EI (2013). Multivariate prediction of subcutaneous glucose concentration in type 1 diabetes patients based on support vector regression. IEEE J. Biomed. Health Informatics.

[22] Gadaleta M, Facchinetti A, Grisan E, Rossi M (2019). Prediction of adverse glycemic events from continuous glucose monitoring signal. IEEE J. Biomed. Health Informatics.

[23] Vehí J, Contreras I, Oviedo S, Biagi L, Bertachi A (2020). Prediction and prevention of hypoglycaemic events in type-1 diabetic patients using machine learning. Health Informatics J..

[24] Dave, D. et al. Feature-based machine learning model for real-time hypoglycemia prediction. *J. Diabetes Sci. Technol.* (2020). 10.1177/1932296820922622 PMC8258517 32476492

[25] Bent B (2021). Engineering digital biomarkers of interstitial glucose from noninvasive smartwatches. npj Digital Med..

[26] Zhu T, Li K, Herrero P, Georgiou P (2021). Deep learning for diabetes: A systematic review. IEEE J. Biomed. Health Informatics.

[27] Fogel AL, Kvedar JC (2018). Artificial intelligence powers digital medicine. npj Digital Med..

[28] Arcadu F (2019). Deep learning algorithm predicts diabetic retinopathy progression in individual patients. npj Digital Med..

[29] Williams BM (2020). An artificial intelligence-based deep learning algorithm for the diagnosis of diabetic neuropathy using corneal confocal microscopy: a development and validation study. Diabetologia.

[30] Zhu T, Li K, Herrero P, Georgiou P (2021). Basal glucose control in type 1 diabetes using deep reinforcement learning: An in silico validation. IEEE J. Biomed. Health Informatics.

[31] Zhu T, Li K, Kuang L, Herrero P, Georgiou P (2020). An insulin bolus advisor for type 1 diabetes using deep reinforcement learning. Sensors.

[32] Sun, Q., Jankovic, M. V., Bally, L. & Mougiakakou, S. G. Predicting blood glucose with an LSTM and Bi-LSTM based deep neural network (2018). 2018 14th Symposium on Neural Networks and Applications (NEUREL).

[33] Zhu, T., Li, K., Herrero, P., Chen, J. & Georgiou, P. A deep learning algorithm for personalized blood glucose prediction (2018). The 3rd International Workshop on Knowledge Discovery in Healthcare Data, IJCAI-ECAI 2018.

[34] Li K, Daniels J, Liu C, Herrero P, Georgiou P (2020). Convolutional recurrent neural networks for glucose prediction. IEEE J. Biomed. Health Informatics.

[35] Li K, Liu C, Zhu T, Herrero P, Georgiou P (2020). GluNet: A deep learning framework for accurate glucose forecasting. IEEE J. Biomed. Health Informatics.

[36] Zhu, T., Li, K., Herrero, P., Chen, J. & Georgiou, P. Dilated recurrent neural networks for glucose forecasting in type 1 diabetes. *J. Healthcare Informatics Res.* 1–17 (2020) . 10.1007/s41666-020-00068-2 PMC8982716 35415447

[37] Deng Y (2021). Deep transfer learning and data augmentation improve glucose levels prediction in type 2 diabetes patients. npj Digital Med..

[38] Zhu, T. et al. IoMT-enabled real-time blood glucose prediction with deep learning and edge computing. *IEEE Internet of Things Journal*10.1109/JIOT.2022.3143375 (2022).

[39] Porumb M, Stranges S, Pescapè A, Pecchia L (2020). Precision medicine and artificial intelligence: a pilot study on deep learning for hypoglycemic events detection based on ECG. Scientific Rep..

[40] Luong, M. T., Pham, H. & Manning, C. D. Effective approaches to attention-based neural machine translation (2015). Conference Proceedings - EMNLP 2015: Conference on Empirical Methods in Natural Language Processing.

[41] Amini, A., Schwarting, W., Soleimany, A. & Rus, D. Deep evidential regression (2020) . Advances in Neural Information Processing Systems .

[42] Finn, C., Abbeel, P. & Levine, S. Model-agnostic meta-learning for fast adaptation of deep networks (2017). Proceedings of the 34th International Conference on Machine Learning.

[43] Spence R, Apperley M (1982). Data base navigation: an office environment for the professional. Behavi. Inform. Technol..

[44] Turksoy K (2013). Hypoglycemia early alarm systems based on multivariable models. Ind. Eng. Chem. Res..

[45] Liu C (2019). Long-term glucose forecasting using a physiological model and deconvolution of the continuous glucose monitoring signal. Sensors.

[46] Preissig CM, Rigby MR (2010). A disparity between physician attitudes and practice regarding hyperglycemia in pediatric intensive care units in the united states: a survey on actual practice habits. Critical Care.

[47] Roberts DR (2017). Cross-validation strategies for data with temporal, spatial, hierarchical, or phylogenetic structure. Ecography.

[48] Bekkink MO, Koeneman M, de Galan BE, Bredie SJ (2019). Early detection of hypoglycemia in type 1 diabetes using heart rate variability measured by a wearable device. Diabetes Care.

[49] Rothberg LJ, Lees T, Clifton-Bligh R, Lal S (2016). Association between heart rate variability measures and blood glucose levels: implications for noninvasive glucose monitoring for diabetes. Diabetes Technol. Ther..

[50] Cichosz SL, Frystyk J, Hejlesen OK, Tarnow L, Fleischer J (2014). A novel algorithm for prediction and detection of hypoglycemia based on continuous glucose monitoring and heart rate variability in patients with type 1 diabetes. J. Diabetes Sci. Technol..

[51] Schuurmans AA (2020). Validity of the empatica e4 wristband to measure heart rate variability (HRV) parameters: a comparison to electrocardiography (ECG). J. Medical Systems.

[52] Hovorka R (2004). Nonlinear model predictive control of glucose concentration in subjects with type 1 diabetes. Physiol. Meas..

[53] Larsen K, Petersen JH, Budtz-Jørgensen E, Endahl L (2000). Interpreting parameters in the logistic regression model with random effects. Biometrics.

[54] Peper, E., Harvey, R., Lin, I.-M., Tylova, H. & Moss, D. Is there more to blood volume pulse than heart rate variability, respiratory sinus arrhythmia, and cardiorespiratory synchrony? Biofeedback 35 (2007) .

[55] Zong, W., Heldt, T., Moody, G. & Mark, R. An open-source algorithm to detect onset of arterial blood pressure pulses (2003). Computers in Cardiology, 2003.

[56] Task Force of the European Society of Cardiology and the North American Society of Pacing and Electrophysiology. Heart rate variability: standards of measurement, physiological interpretation and clinical use. *Circulation* **93**, 1043–1065 (1996). 8598068

[57] Benedek M, Kaernbach C (2010). A continuous measure of phasic electrodermal activity. J. Neurosci. Methods.

[58] Carreiras, C. et al. BioSPPy: Biosignal processing in Python https://github.com/PIA-Group/BioSPPy/ (2015).

[59] Makowski, D. et al. Neurokit2: A Python toolbox for neurophysiological signal processing https://github.com/neuropsychology/NeuroKit (2020). 10.3758/s13428-020-01516-y 33528817

[60] Marling, C., Xia, L., Bunescu, R. & Schwartz, F. Machine learning experiments with noninvasive sensors for hypoglycemia detection (2016). *Proceedings of IJCAI Workshop on Knowledge Discovery in Healthcare Data*. Morgan Kaufmann Publishers Inc., San Francisco, CA, USA.

[61] Zisser H (2008). Bolus calculator: a review of four “smart” insulin pumps. Diabetes Technol. Ther..

[62] Toloşi L, Lengauer T (2011). Classification with correlated features: unreliability of feature ranking and solutions. Bioinformatics.

[63] Li L, Jamieson K, DeSalvo G, Rostamizadeh A, Talwalkar A (2017). Hyperband: a novel bandit-based approach to hyperparameter optimization. J. Machine Learning Res..

[64] Nichol, A., Achiam, J. & Schulman, J. On first-order meta-learning algorithms Preprint at https://arxiv.org/abs/1803.02999 (2018).

[65] Raghu, A., Raghu, M., Bengio, S. & Vinyals, O. *Rapid learning or feature reuse? towards understanding the effectiveness of MAML* (2019). International Conference on Learning Representations.

[66] Zhu, T., Yao, X., Li, K., Herrero, P. & Georgiou, P. *Blood glucose prediction for type 1 diabetes using generative adversarial networks* (2020). The 5th International Workshop on Knowledge Discovery in Healthcare Data, ECAI 2020.

[67] Hochreiter S, Schmidhuber J (1997). Long short-term memory. Neural Comput..

[68] Chung, J., Gulcehre, C., Cho, K. & Bengio, Y. *Empirical evaluation of gated recurrent neural networks on sequence modeling* (2014). NIPS 2014 Workshop on Deep Learning, December 2014.

[69] Schuster M, Paliwal KK (1997). Bidirectional recurrent neural networks. IEEE Trans. Signal Process..

[70] Bahdanau, D., Cho, K. & Bengio, Y. Neural machine translation by jointly learning to align and translate (2015). 3rd International Conference on Learning Representations, ICLR.

[71] Del Favero S, Facchinetti A, Cobelli C (2012). A glucose-specific metric to assess predictors and identify models. IEEE Trans. Biomed. Eng..

[72] Danne T (2017). International consensus on use of continuous glucose monitoring. Diabetes Care.

[73] Chicco D, Jurman G (2020). The advantages of the matthews correlation coefficient (MCC) over F1 score and accuracy in binary classification evaluation. BMC Genomics.

[74] Herrero P (2012). Robust fault detection system for insulin pump therapy using continuous glucose monitoring. J. Diabetes Sci. Technol..

[75] Dalla Man C (2014). The UVA/PADOVA type 1 diabetes simulator: new features. J. Diabetes Sci. Technol..

[76] Liu C (2020). A modular safety system for an insulin dose recommender: a feasibility study. J. Diabetes Sci. Technol..

