# Supplementary material for: Enhancing self-management in type 1 diabetes with wearables and deep learning
Source: NPJ Digit Med. 2022 Jun 27;5:78. doi: 10.1038/s41746-022-00626-5 (PMC9237131; doi:10.1038/s41746-022-00626-5)
Supplement: Supplementary file 1 — Reporting Summary [file 41746_2022_626_MOESM1_ESM.pdf]

nature portfolio

Corresponding author(s): Taiyu Zhu, Kezhi Li

Last updated by author(s): Aug 16, 2021

Reporting Summary

Nature Portfolio wishes to improve the reproducibility of the work that we publish. This form provides structure for consistency and transparency in reporting. For further information on Nature Portfolio policies, see our Editorial Policies and the Editorial Policy Checklist.

Statistics

For all statistical analyses, confirm that the following items are present in the figure legend, table legend, main text, or Methods section.

n/a	Confirmed	
☐	☒	The exact sample size (*n*) for each experimental group/condition, given as a discrete number and unit of measurement
☐	☒	A statement on whether measurements were taken from distinct samples or whether the same sample was measured repeatedly
☐	☒	The statistical test(s) used AND whether they are one- or two-sided<br>*Only common tests should be described solely by name; describe more complex techniques in the Methods section.*
☐	☒	A description of all covariates tested
☐	☒	A description of any assumptions or corrections, such as tests of normality and adjustment for multiple comparisons
☐	☒	A full description of the statistical parameters including central tendency (e.g. means) or other basic estimates (e.g. regression coefficient) AND variation (e.g. standard deviation) or associated estimates of uncertainty (e.g. confidence intervals)
☐	☒	For null hypothesis testing, the test statistic (e.g. *F*, *t*, *r*) with confidence intervals, effect sizes, degrees of freedom and *P* value noted<br>*Give P values as exact values whenever suitable.*
☒	☐	For Bayesian analysis, information on the choice of priors and Markov chain Monte Carlo settings
☒	☐	For hierarchical and complex designs, identification of the appropriate level for tests and full reporting of outcomes
☒	☐	Estimates of effect sizes (e.g. Cohen's *d*, Pearson's *r*), indicating how they were calculated

Our web collection on statistics for biologists contains articles on many of the points above.

Software and code

Policy information about availability of computer code

Data collection	The data was collected by the daily entries of smartphone app and the reporting software of Dexcom G6 and Empatica E4.
Data analysis	The data was analyzed by R 3.6.3 and Python 3.8.5.

For manuscripts utilizing custom algorithms or software that are central to the research but not yet described in published literature, software must be made available to editors and reviewers. We strongly encourage code deposition in a community repository (e.g. GitHub). See the Nature Portfolio guidelines for submitting code & software for further information.

Data

Policy information about availability of data

All manuscripts must include a data availability statement. This statement should provide the following information, where applicable:

- Accession codes, unique identifiers, or web links for publicly available datasets
- A description of any restrictions on data availability
- For clinical datasets or third party data, please ensure that the statement adheres to our policy

The dataset used in this study is not publicly available due to the proprietary nature of the data and privacy concerns. Interested researchers should contact the corresponding authors to inquire about the access.

Field-specific reporting

Please select the one below that is the best fit for your research. If you are not sure, read the appropriate sections before making your selection.

☒ Life sciences ☐ Behavioural & social sciences ☐ Ecological, evolutionary & environmental sciences

For a reference copy of the document with all sections, see nature.com/documents/nr-reporting-summary-flat.pdf

Life sciences study design

All studies must disclose on these points even when the disclosure is negative.

Sample size	The data were collected from 12 adults with type 1 diabetes over a six-week clinical trial. For each subject, a median (IQR) of 1,113.5 (1,059.0-1,184.0) and 832.5 (733.0-953.0) hours of glucose data and sensor wristband data were available for model development.
Data exclusions	No data were excluded. The analysis was performed on each subject.
Replication	The model can reproduce identical results as those reported in the article using the same weights and input features.
Randomization	This is not applicable to this study since there was not an intervention.
Blinding	This is not applicable to this study since there was not an intervention.

Reporting for specific materials, systems and methods

We require information from authors about some types of materials, experimental systems and methods used in many studies. Here, indicate whether each material, system or method listed is relevant to your study. If you are not sure if a list item applies to your research, read the appropriate section before selecting a response.

Materials & experimental systems

n/a	Involved in the study	
☒	☐	Antibodies
☒	☐	Eukaryotic cell lines
☒	☐	Palaeontology and archaeology
☒	☐	Animals and other organisms
☐	☒	Human research participants
☒	☐	Clinical data
☒	☐	Dual use research of concern

Methods

n/a	Involved in the study	
☒	☐	ChIP-seq
☒	☐	Flow cytometry
☒	☐	MRI-based neuroimaging

Human research participants

Policy information about studies involving human research participants

Population characteristics	12 participants with type 1 diabetes, aged 30-50; 6 female, 6 male; 6 used multiple daily injection, 6 used continuous subcutaneous insulin infusion.
Recruitment	All participants were recruited from the Imperial College Healthcare NHS trust T1DM outpatient clinics, registered research databases, and interested participants who contact us. All participant visits were conducted within the clinical research unit at the NIHR/Wellcome Trust Imperial Clinical Research Facility.
Ethics oversight	The trial protocol was approved by the Medicine and Healthcare Products Regulatory Agency (MHRA).

Note that full information on the approval of the study protocol must also be provided in the manuscript.
