# Supplementary material for: Enhancing self-management in type 1 diabetes with wearables and deep learning
Source: NPJ Digit Med. 2022 Jun 27;5:78. doi: 10.1038/s41746-022-00626-5 (PMC9237131; doi:10.1038/s41746-022-00626-5)
Supplement: Supplementary file 2 — Supplementary Information [file 41746_2022_626_MOESM2_ESM.pdf]

Supplementary information: Enhancing self-management in type 1 diabetes with wearables and deep learning

Taiyu Zhu*, Chukwuma Uduku, Kezhi Li*, Pau Herrero, Nick Oliver and Pantelis Georgiou

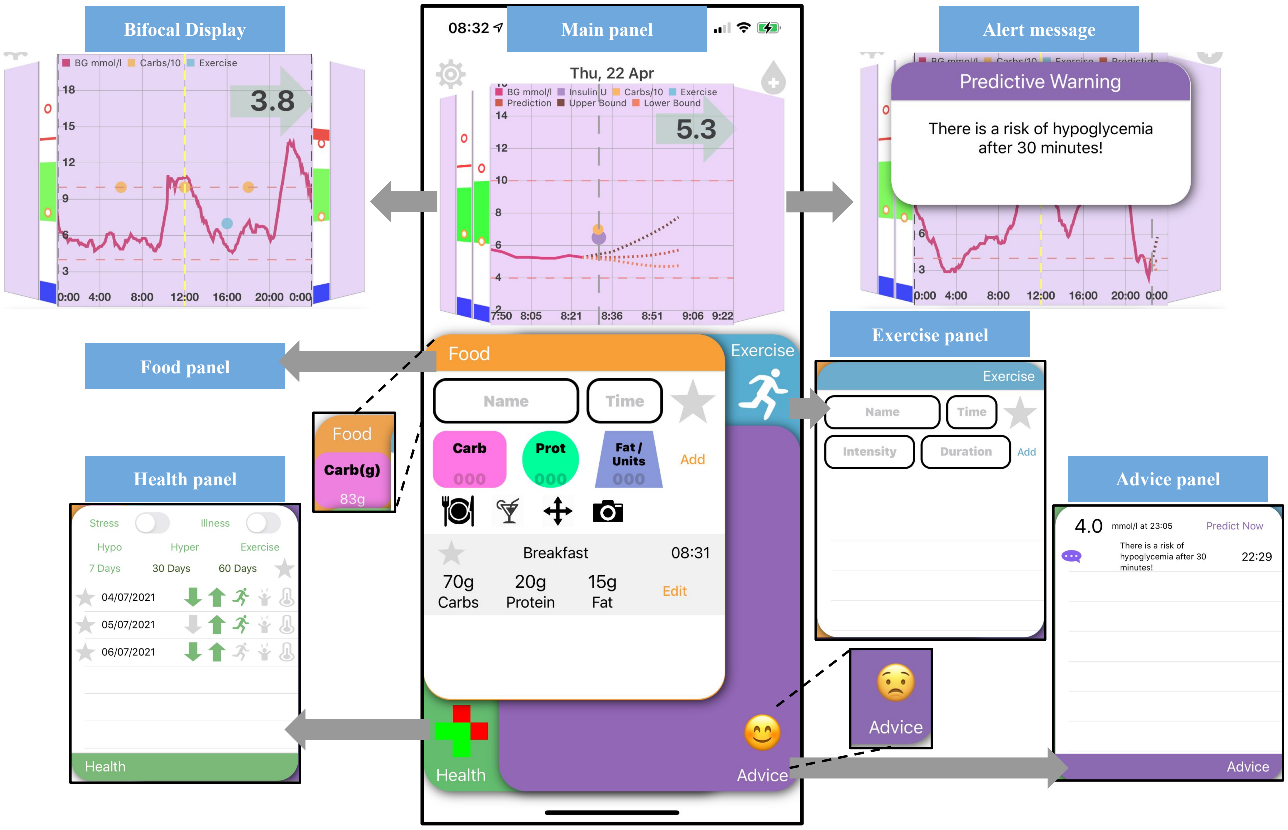

Supplementary Figure 1: Graphical user interface of the ARISES app, where bifocal display is used to present glucose trajectories. The users are allowed to record daily activities through the food, exercise and health panels. The embedded deep learning algorithm predicts glucose levels and detects hypo- and hyperglycemia with lower and upper bounds. Then the app sends corresponding warnings to users by notifications and haptic vibration and displays information in the advice panel. The corner icons of the food and advice panels can show the details of carbohydrate and the status of unread alert messages, respectively, when these panels are stacked at the bottom.

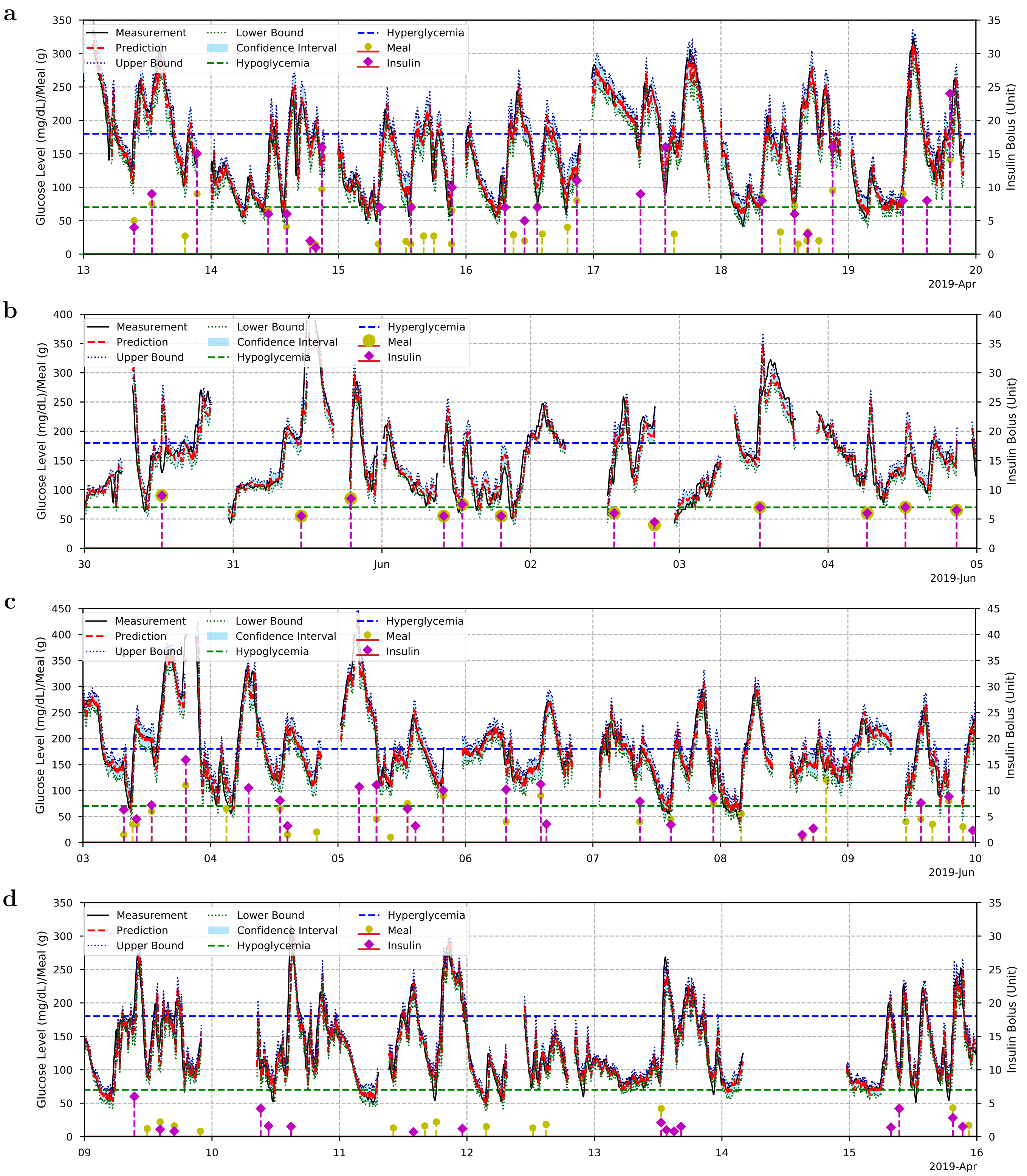

Supplementary Figure 2: Seven-day period CGM and prediction trajectories of T1D adults over a 30-minute prediction horizon, each representing one of the four subgroups in the clinical study. a a male participant with MDI regimen. **b** a female participant with MDI regimen. **c** a male participant with CSII regimen. **d** a female participant with CSII regimen.

Supplementary Table 1: Number of daily entries manually recorded by the participants in the phase I of the ARISES clinical study

**Entries**	**All (n=5767)**		**MDI (n=2988)**		**CSII (n=2779)**	
	% (n)	Median (IQR)	% (n)	Median (IQR)	% (n)	Median (IQR)
Carbohydrates	39.6% (2285)	207 (122-249.5)	41.2% (1233)	172 (127.5-285.5)	37.9% (1052)	213 (107.5-242)
Protein	16.9% (976)	132 (33-163)	17.5% (522)	42 (4-146)	16.3% (454)	158 (138-168)
Fat	16.5% (952)	135 (31-163)	16.5% (492)	40 (4-146)	16.5% (460)	158 (140.5-168.5)
Insulin Bolus	20.6% (1189)	111 (57-142.5)	18.9% (565)	101 (50-131)	22.5% (624)	123 (77.5-144.5)
Exercise	4.5% (262)	19.5 (13-29.5)	4.9% (148)	19.5 (13.8-28.3)	4.1% (114)	18.5 (13.5-27.3)
Alcohol	1.2% (72)	6 (4-9)	0.5% (14)	2.5 (0.75-5.25)	2.1% (58)	8 (6-17)
Stress	0.3% (18)	2.5 (2-3)	0.1% (4)	2 (2-2)	0.5% (14)	3 (2.5-4)
Illness	0.3% (18)	3 (3-4.8)	0.3% (10)	10 (10-10)	0.3% (8)	3 (2.5-3)

Supplementary Table 2: Information of daily entries manually recorded by the participants (Median (IQR)) in the phase I of the ARISES clinical study

**Entries**	**All**	**Baseline**	**Endpoint**	p
Total daily carbohydrate (grams)	160 (102-220)	160 (100-228)	145 (101-220)	0.77
Breakfast carbohydrate (grams)	41 (25-60)	40 (26-60)	42 (29.3-58.8)	0.68
Lunch carbohydrate (grams)	45 (30-60)	50 (33.8-62.8)	40 (30-65)	0.14
Dinner carbohydrate (grams)	50 (40-70)	50 (40-70)	50 (40-75)	0.50
Daily Bolus insulin (units)	20 (14.8-28)	20.2 (15.9-28.6)	20 (14-27.5)	0.29
Number exercise	19.5 (11.8-30.3)	8.5 (7.8-12.3)	7 (2.3-8)	0.10
Daily engagement	7 (4-9)	7 (4.8-9)	6 (4-8)	0.08
Number of interactions	396 (237-732.3)	152 (121-255.3)	127 (89-229.8)	0.42

Supplementary Table 3: Description of features

**Wristband (Empatica E4)**	
IBI	Inter-beat intervals
meanHR	Mean heart rate
medianNNI	Median value of normal-to-normal (NN) intervals
SDNN	Standard deviation of NN intervals
RMSSD	Root mean square of successive differences between adjacent NNs
CVSD	Coefficient of variation of successive differences
CVNNI	Coefficient of variation of NN intervals
pNNX	Percentage of successive NN intervals greater than 50 ms
LHR	Low-/high-frequency power ratio
EDA	electrodermal activity
SCL	Skin conductance level
SCR	Skin conductance response
ACC	Average 3-D acceleration
TEMP	Skin temperature
**CGM (Dexcome G6)**	
CGM	CGM measurement sequence
timeIndex	Normalized time sequence
**Daily entries**	
Carb	Amount of carbohydrate intake
IOB	Insulin on board
Bolus	Amount of insulin bolus delivery
COB	Carbohydrate on board

Supplementary Table 4: Performance of the baseline methods for glucose level prediction over the 60-minute prediction horizon

Methods	RMSE (mg/dL)	gRMSE (mg/dL)	MAE (mg/dL)	MAPE (%)	Time lag (min)
PKM	$43.12 \pm 7.53^{**}$	$51.22 \pm 10.42^{**}$	$31.51 \pm 5.09^{**}$	$21.95 \pm 4.28^{**}$	$19.87 \pm 10.26^{*}$
ARMA	$42.29 \pm 7.73^{**}$	$53.44 \pm 10.24^{**}$	$30.77 \pm 5.43^{**}$	$21.04 \pm 3.96^{**}$	$41.89 \pm 13.19^{**}$
SVR	$39.99 \pm 7.03^{**}$	$52.03 \pm 10.48^{**}$	$31.52 \pm 5.93^{**}$	$24.19 \pm 5.61^{**}$	21.34 ± 11.49
ANN	$39.31 \pm 6.85^{**}$	$51.88 \pm 9.59^{**}$	$29.69 \pm 5.35^{**}$	$21.15 \pm 4.10^{**}$	$31.88 \pm 13.65^{**}$
Bi-LSTM	$39.32 \pm 7.19^{**}$	$51.62 \pm 9.92^{**}$	$29.02 \pm 5.23^{**}$	$20.21 \pm 4.01^{**}$	19.57 ± 10.71
CRNN	$37.18 \pm 6.09^{**}$	$49.04 \pm 8.50^{**}$	$27.77 \pm 4.89^{**}$	$19.55 \pm 3.70^{**}$	21.57 ± 11.41

Root mean square error (RMSE), glucose-specific RMSE (gRMSE), mean absolute error (MAE), mean absolute percent error (MAPE), and the time lag are employed as metrics. The significance is indicated as $^{*}p < 0.05$, $^{**}p < 0.01$.

Supplementary Table 5: Performance of the baseline methods for hypoglycemia prediction over the 60-minute prediction horizon

Methods	Accuracy (%)	Sensitivity (%)	Specificity (%)	Precision (%)	MCC score	MD (mg/dL)
PKM	$80.05 \pm 6.29^{**}$	$82.49 \pm 13.10^{*}$	$78.75 \pm 8.77^{**}$	$36.43 \pm 12.98^{**}$	$0.45 \pm 0.11^{*}$	$40.36 \pm 15.34^{**}$
ARMA	91.89 ± 5.23	$56.95 \pm 19.24^{**}$	94.87 ± 3.67	55.28 ± 17.38	0.51 ± 0.12	$41.92 \pm 14.60^{**}$
SVR	87.20 ± 6.08	$6.03 \pm 10.16^{**}$	$99.10 \pm 1.40^{**}$	30.05 ± 32.98	$0.08 \pm 0.12^{**}$	$60.55 \pm 22.73^{**}$
ANN	88.01 ± 5.80	$17.15 \pm 11.75^{**}$	$98.39 \pm 1.43^{**}$	61.76 ± 15.16	$0.26 \pm 0.11^{**}$	$48.18 \pm 18.05^{**}$
Bi-LSTM	87.51 ± 6.65	$19.78 \pm 12.38^{**}$	$97.52 \pm 1.92^{*}$	47.79 ± 20.69	$0.25 \pm 0.15^{**}$	$43.61 \pm 16.73^{**}$
CRNN	88.06 ± 5.94	$12.54 \pm 8.40^{**}$	$99.07 \pm 0.72^{**}$	67.70 ± 16.88	$0.24 \pm 0.11^{**}$	$45.94 \pm 19.37^{**}$

MCC: Matthews correlation coefficient. MD: mean deviation from true glucose levels for missed predicted hypoglycemic events. The significance is indicated as $^{*}p < 0.05$, $^{**}p < 0.01$.

Supplementary Table 6: Performance of the baseline methods for hyperglycemia prediction over the 60-minute prediction horizon

Methods	Accuracy (%)	Sensitivity (%)	Specificity (%)	Precision (%)	MCC score	MD (mg/dL)
PKM	85.54 ± 3.13	91.58 ± 3.52	$77.21 \pm 4.74^{*}$	$79.68 \pm 12.49^{*}$	0.68 ± 0.06	$61.42 \pm 16.26^{**}$
ARMA	$85.05 \pm 1.85^{**}$	$82.26 \pm 7.02^{**}$	83.52 ± 6.27	84.59 ± 6.51	$0.66 \pm 0.04^{**}$	$65.36 \pm 14.47^{**}$
SVR	$84.73 \pm 3.24^{**}$	83.44 ± 15.68	75.59 ± 19.79	84.04 ± 5.22	$0.64 \pm 0.08^{**}$	$52.09 \pm 10.85^{*}$
ANN	$84.13 \pm 2.15^{**}$	$76.24 \pm 18.61^{*}$	82.43 ± 13.46	85.77 ± 4.85	$0.62 \pm 0.08^{**}$	$56.65 \pm 11.12^{**}$
Bi-LSTM	$84.22 \pm 3.38^{**}$	$77.03 \pm 17.55^{*}$	83.19 ± 11.59	85.84 ± 4.58	$0.63 \pm 0.08^{**}$	$57.43 \pm 10.60^{**}$
CRNN	$83.97 \pm 4.33^{**}$	$76.03 \pm 20.07^{*}$	82.70 ± 14.53	87.28 ± 4.57	$0.63 \pm 0.09^{**}$	$53.47 \pm 10.27^{*}$

MCC: Matthews correlation coefficient. MD: mean deviation from true glucose levels for missed predicted hyperglycemic events. The significance is indicated as $^{*}p < 0.05$, $^{**}p < 0.01$.

Supplementary Table 7: Hyperparameters used for the proposed deep learning model

Hyperparameter	Value
Hidden units of the Bidirectional GRU layer	128
Hidden units of the GRU layer	128
Hidden units of the attention layer	128
Learning rate of meta-learning	1e-4
Learning rate of fine-tuning	1e-5
Batch size	64
Number of epochs	500
Early stopping patience	50
Length of sequence	12
Feature Dimension	6
