# Supplementary material for: Enhancing self-management in type 1 diabetes with wearables and deep learning
Source: NPJ Digit Med. 2022 Jun 27;5:78. doi: 10.1038/s41746-022-00626-5 (PMC9237131; doi:10.1038/s41746-022-00626-5)
Supplement: Supplementary file 3 — Clinical Trial Protocol [file 41746_2022_626_MOESM3_ESM.pdf]

Clinical Trial Protocol

Adaptive, Real-time, Intelligent System to Enhance Self-care of chronic disease (ARISES)

Chief investigator: **Professor Nick Oliver**
Co-Investigators: **Dr Chukwuma Uduku, Dr Monika Reddy, Dr Pantelis Georgiou, Dr Pau Herrero, Prof Robert Spence, Dr Kezhi Li, Mr John Daniels**

General information

**Title:**	Adaptive, Real-time, Intelligent System to Enhance Self-care of chronic diseases (ARISES)
**Identifying Number:**	Imperial College Joint Research Office – 18HH4410
**Date:**	Version 1.0 18/12/2017
**Sponsor:**	Imperial College London<br>Joint Research Compliance Office<br>Imperial College London and Imperial College Healthcare NHS Trust<br>Room 215, Level 2 Medical School Building<br>Norfolk Place<br>London, W2 1PG
**Sponsor Contact:**	Becky Ward<br>Research Governance Manager<br>Joint Research Compliance Office<br>Imperial College London and Imperial College Healthcare NHS Trust<br>Room 215, Level 2 Medical School Building<br>Norfolk Place, London, W2 1PG<br>Email: becky.ward@imperial.ac.uk<br>Telephone: 0207 594 9459
**Chief Investigator (clinical):**	Nick Oliver<br>St. Mary's Hospital Medical School Building,<br>Imperial College, London, W2 1PG, UK<br>Nick.oliver@imperial.ac.uk<br>0207 594 2460

**Protocol Authorised by:**	Name & Role	Date	Signature

Clinical Queries: Clinical queries should be directed to Dr Chukwuma Uduku who will direct the query to the appropriate person

Funder: The project is being funded by an Engineering and Physical Sciences Research Council (EPSRC) grant

This protocol describes the **ARISES** study and provides information about procedures for entering participants. Every care was taken in its drafting, but corrections or amendments may be necessary. These will be circulated to investigators in the study. Problems relating to this study should be referred, in the first instance, to the Chief Investigator.

This study will adhere to the principles outlined in the NHS Research Governance Framework for Health and Social Care (2nd edition). It will be conducted in compliance with the protocol, the Data Protection Act and other regulatory requirements as appropriate.

Contents

1.0 Introduction
1.1 Background 4
1.2 Components: 6
1.3 Safety measures: 7

2.0 Study objectives and design 8
2.1 Focus Groups 8
Objectives 8
Timescale 8
Recruitment 8
Format 8
2.2 Clinical studies 9
2.2.1 Physiological and environmental data collection 9
Objectives 10
Methods 10
Recruitment 10
Inclusion criteria 10
Exclusion criteria 10
Withdrawal criteria 11
Screening visit 11
Assessment and follow-up 12
Statistics and data analysis 13
3.0 Safety 14
Adverse event definitions 14
Adverse event reporting procedures 14
4.0 Regulatory Issues 15
Ethics Approval 15
Consent 15
Confidentiality 15
Indemnity 15
Sponsor 16
Funding 16
Audits 16

5.0 Study Management 16

6.0 Publication Policy 16

1.1 Background

Diabetes affects more than 3 million UK inhabitants with a global prevalence that has almost doubled since 1980[1]. Type 1 diabetes (T1DM) accounts for 10% of the UK diabetes population and is characterised by insulin deficiency and impaired glucose homeostasis secondary to autoimmune destruction of pancreatic beta cells[2] (Diabetes UK: Annual report 2016 https://diabetes-resources-production.s3-eu-west-1.amazonaws.com/diabetes-storage/2017-08/Annual_Report_2016.pdf).

Therapeutic insulin replacement aims to replicate physiological insulin secretion in response to glycaemia. The most accessible mode of insulin delivery is by multiple daily subcutaneous injections (MDI). Alternatively, continuous subcutaneous insulin infusion (CSII) via an insulin pump delivers variable basal insulin rates throughout the day with the benefit of fewer injections.

There is evidence to advocate intensive insulin therapy to limit blood glucose excursions and reduce the risk of microvascular complications[3]. However, intensive insulin therapy is associated with an increased risk of recurrent hypoglycaemia and in severe cases its dangerous sequalae of seizures, coma and death[456]. Subcutaneous continuous glucose monitoring (CGM) has been shown to help improve overall glycaemic control and reduce the incidence of hypoglycaemia[7].

Insulin bolus calculators have been developed to help overcome hypoglycaemia and post prandial excursions associated with insulin carbohydrate mismatch[8,9]. Most insulin pump devices and a growing number of capillary glucose meters have insulin bolus calculators integrated into the hardware. Standard bolus calculations are derived from a formula requiring current blood glucose levels, target blood glucose, total carbohydrate in meals (grams), insulin-to-carbohydrate ratio (ICR, grams of CHO per 1 unit of insulin), insulin sensitivity factor (ISF, glucose reduction per 1 unit of insulin) and insulin on board (IOB, active insulin from previous bolus administrations). Structured education remains an important part of empowering self-management in T1DM by encouraging appropriate self-glucose monitoring, carbohydrate (CHO) counting and insulin dose-adjustment[10].

Over 15 million people in the UK have a long-term chronic illness and are at risk of the physical and psychosocial ramifications that arise with sub-optimal management (Department of Health (2012). Report. Long-term conditions compendium of Information: 3rd edition https://www.gov.uk/government/uploads/system/uploads/attachment_data/file/216528/dh_134486.pdf). In the case of diabetes, associated complications such as ischaemic heart disease, stroke, blindness and disability following limb amputation all carry a significant socioeconomic burden. Effective diabetes self-management has been shown to reduce the risk of complications.

The Adaptive, Real-time, Intelligent System to Enhance Self-care of chronic diseases (ARISES) is a novel mobile platform capable of collecting data from multiple sources to empower people to more effectively self-manage chronic disease through therapeutic and lifestyle decision support (Figure 1). Using wearable sensors and smartphone technology, a wide range of biological, environmental and behavioural data will feedback into the system to facilitate local real-time decision support and adaptive personalised care using machine learning and Case-Based Reasoning (CBR) (Figure 2). CBR is an artificial intelligence methodology capable of solving problems by referring to historic data and outcomes from previously encountered scenarios (cases). A case consists of the problem description (e.g.

exercise), the solution (recommended snack) and the outcome (blood glucose post exercise). Automated (e.g. ambient temperature) and manual (e.g. meal macronutrient content) parameter inputs will be fed into the CBR algorithm as case problems. The algorithm proceeds to filter through previously encountered scenarios to **retrieve** a similar case and **adapt** the case information to recommend an improved solution. Proposed solutions are **evaluated**, and potentially useful outcomes are **stored** in the case base. The ARISES CBR algorithm will run locally on a smartphone device. The system will be designed to offer decision support applicable and specific to patients receiving MDI and CSII methods of insulin delivery.

Figure 1: Illustration of the ARISES concept

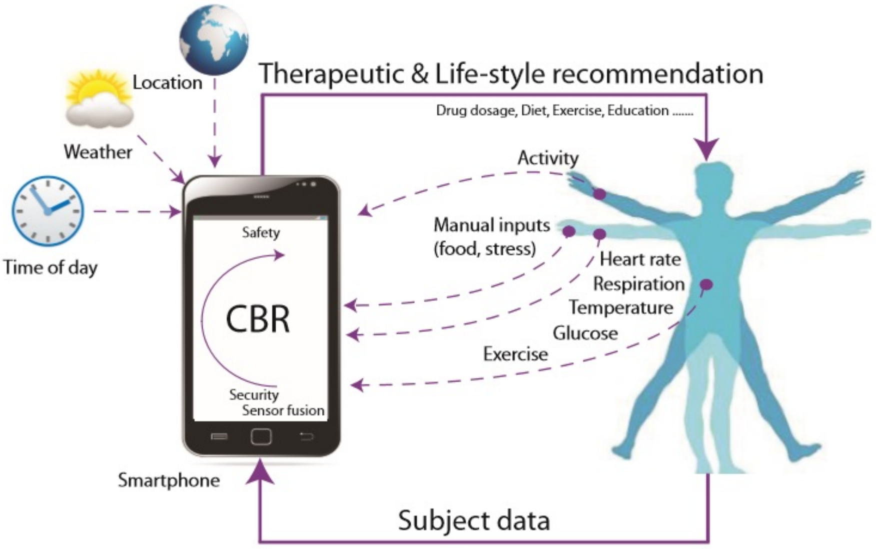

Figure 1: ARISES concept

Figure 2: The CBR cycle

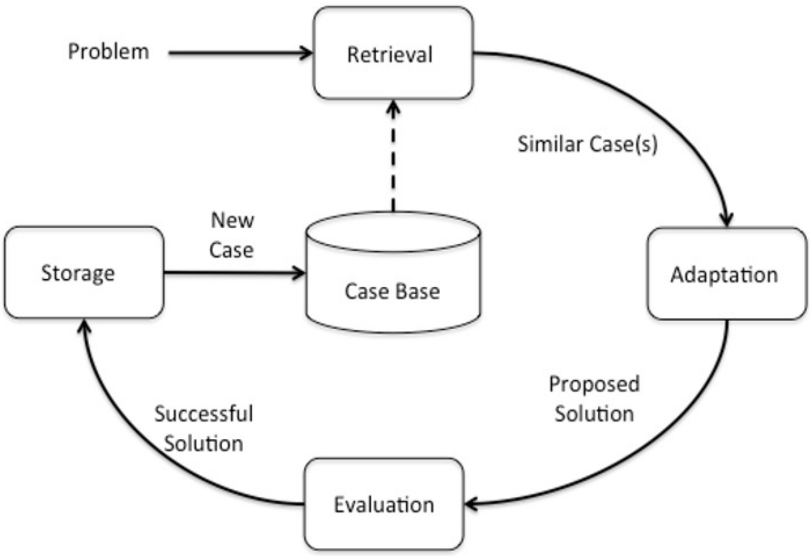

ARISES aims to use participants with T1DM as an exemplar case study. The system will target self-management to optimise glucose control through insulin dose recommendation (therapeutic advice), exercise and physical stress support, hypoglycaemia prevention through timely carbohydrate and snack recommendation and behavioural change through educational (lifestyle advice). Measures to safeguard patients will be ensured through education, predictive glucose alarm and carbohydrate recommendation. Patients with T1DM will be recruited as part of the design team to offer feedback on user requirements and ergonomic interface development throughout the project.

This protocol details the semi-structured focus groups aimed at designing a user friendly ARISES mobile platform. Focus groups will be comprised of investigators and patients with T1DM. The protocol also outlines a six-week observational clinical study (phase 1) aiming to monitor and assess key inputs affecting glycaemia in patients with T1DM. Physiological and environmental data parameters will be assessed using wearable technologies and manual data will be logged by the participant into a mobile app. Data drawn from the study will serve as a training set to assist the development of CBR artificial intelligence to be integrated into the completed ARISES platform. ARISES is a feasibility study beyond the work outlined in this protocol. The project requires the outputs derived from the observational study and focus groups for final study design (phase 2).

1.2 Components of data collection study (phase 1)

Continuous glucose monitoring

The Dexcom glucose sensor (CE marked and a licensed medical device in the UK, manufactured by Dexcom) will be used throughout the clinical trials. The sensor is deployed subcutaneously and samples interstitial fluid using an enzyme electrode. A small voltage is applied across the sensor and a current is fed back to the sensor instrumentation. This current is proportional to the glucose concentration in interstitial fluid and is calibrated against blood glucose a minimum of 12-hourly. Dexcom CGM data will be transmitted and stored wirelessly to a mobile smart phone device and uploaded to a secure cloud-based server using the Diasend app. Participants will be non-blinded to CGM data throughout the clinical studies.

Insulin delivery system

MDI – Participants on MDI will continue using the same insulin therapy they were on and inject into subcutaneous tissue.

CSII – Participants using CSII will continue using the pump device and basal insulin rate profiles they were on. The pump will deliver insulin from a cartridge reservoir through cannula tubing and into subcutaneous tissue.

Sensor devices

Empatica E4 – Real time physiological data will be recorded continuously using the Empatica E4 wireless multi-sensor wristband. The Empatica E4 wristband houses a photoplethysmography (PPG) sensor capable of monitoring heart rate and heart rate variability; an electrodermal activity (EDA) sensor used to measure sympathetic activity in response to activity and stressful stimuli; an infrared thermophile for measuring peripheral skin temperature; and a 3-axis accelerometer to detect motion-based activity. All the above data parameters will be uploaded to a secure proprietary cloud-based platform via a wired connection to a personal computer.

Smartphone – Apple iPhone devices will monitor and locally store location and ambient temperature data using built in GPS and WIFI technology. Using the myTracks app, these parameters will be retrieved retrospectively to assess their impact on glycaemia. Manual data inputs such as insulin dose, meal macronutrient composition in grams and exercise will be logged by the participant using the mySugr mobile application.

1.3 Safety measures

Hypo and Hyperglycaemia alarms
The Dexcom mobile CGM system will send an audio alarm to the user's smartphone or receiver to alert them of impending hypo and hyperglycaemia. This will enable participants to bring glucose levels back to target range in a timely fashion and limit periods spent in extreme glycaemia.

Fault detection system
The Dexcom mobile CGM system will identify and send an audio alarm to the user's smartphone or receiver to alert them of hardware faults.

Hypoglycaemia management education
Participants will receive formal education and assessment on hypoglycaemia management during screening visit.

2.0. Study Objectives and Design

The key research objectives are to assesses the usability, safety and proof of concept of ARISES in chronic disease using participants with T1DM as an exemplar case study.

This protocol describes the semi-structured focus groups aimed at designing a user friendly ARISES mobile platform and a six-week observational study (phase 1) to monitor and evaluate key inputs affecting glycaemia using wearable technologies. Safety will be assessed throughout the clinical studies. Dexcom RT-CGM will be used throughout all clinical trial phases.

2.1 Focus Groups

Semi-structured focus groups made up of patients with T1DM, and observed by clinicians, engineers and experts in human computer interaction will provide a forum to gather feedback and ideas on data representation and presentation. The meetings will also allow an opportunity to acquire patient feedback on clinical study protocols and logistics.

Objectives:

- Establish important usability requirements to incorporate into the ARISES mobile interface design.
- Collaboratively design a user friendly mobile application interface that adheres to standard procedures using human computer interaction and follows relevant standards (IEC 62366).
- Design will focus on intuitive and easy access to decision support with as few device interactions as possible whilst maintaining sight of real time outcomes on glycaemia.

Timescale: Twelve 2-hourly focus meetings will be held over the entire course of the study.

Participants: 10 participants with T1DM will be expected to attend at least 6 focus groups. Meetings will be observed by a diabetes specialist clinician, engineers and experts in human computer interaction.

Recruitment: Participants with T1DM will be recruited from the Imperial College Healthcare NHS trust type 1 diabetes outpatient clinics, from registered research databases and from interested participants who contact us.

Format: Focus group discussions will be chaired by a diabetes specialist clinician. The specific objectives for each meeting will vary depending on design ideas and issues encountered following feedback from previous focus group meetings.

Focus groups will be interactive and follow a semi-structured format as outlined below:

- A questionnaire with topics for discussion will be sent to participants in advance.
- 15-minute presentation by a member of the investigative team to illustrate current design ideas and concepts.
- 40-minutes focused discussion and feedback on presentation objectives
- 40-minutes focussed discussion on questionnaire.
- 20-minutes open floor discussion.

- 5- minutes conclusion.

Responses to planned questions, additional ideas and points of discussion will be documented by an attending member of the investigative team on a prepared template. With agreed consent and anonymity respected, useful quotes will be taken from participants.

Questionnaires and templates will vary for each meeting depending on newly conceived interface ideas and the outcomes from previous focus meetings.

Outcomes from each focus group meeting, including collected participant questionnaire data, will be anonymously shared among all attendees for validation. Outcomes will subsequently be discussed among the investigative team and where appropriate considered for integration in the mobile interface design.

2.2. Clinical studies

2.2.1 Clinical physiological and environmental data collection

This first phase clinical study protocol will serve as a training set for data collection and evaluation of blood glucose correlations against measured physiological and environmental case parameters. During this study glucose levels will be monitored continuously using RT CGM alongside various physiological parameters (see **Table 1**) using the Empatica E4 wristband. Using the myTracks app, location and ambient temperature data will be automatically logged and retrospectively accessed from iPhone devices either owned by or issued to participants during the study. Data requiring manual input (e.g. exercise, meal macronutrients and alcohol intake) will be logged in the mySugr app installed on the participant's smartphone for retrospective analysis. Participants will have access to CGM data which they will be supported to use in their self-management and they will continue with their normal insulin regimen during this phase.

Issued iPhone devices and the Empatica wristbands will be returned at the end of the study.

Table 1

**Automated inputs (Empatica E4 wristband)**	**Manually entered inputs (mySugr App)**	**Retrospectively sourced inputs (myTracks app)**
Heart rate	Meals – time and macronutrient composition (carbohydrate, protein and fat)	Geolocation
Blood volume pulse	Alcohol – time and amount in units	Ambient temperature
Peripheral skin temperature	Exercise – time, duration and type (intensity)	
Motion activity	Intercurrent stress	
Sympathetic activity		
Electrodermal activity/Galvanic skin response		

Objective - Derive useful blood glucose correlations against measured parameters to incorporate into CBR algorithm development and important wearable technology to maximise use and outcomes in the final ARISES platform feasibility study (phase 2).

Methods – Non-randomised open study

Timescale – Each participant will be in the study for 6 weeks, with a total of 3 visits in that timeframe. The availability of six Empatica E4 wristbands to be shared among 12 participants obligates the study to last for a total of 12 weeks.

Primary outcome – This clinical study will be a data collection exercise with no set primary outcomes. Nonetheless, this study can provide questionnaire and feasibility outcomes for combining both CGM and a physiological data acquisition sensor.

Recruitment – Participants for all the clinical studies will be recruited from the Imperial College Healthcare NHS trust T1DM outpatient clinics, from registered research databases and from interested participants who contact us. Participant information sheets will be given to potential participants and, after a minimum of 48 hours and following any questions, informed consent will be taken.

Participants – Twelve adults with T1DM

Participant inclusion criteria

- Adults ≥18years of age
- Diagnosis of T1DM for > 1 year
- Structured education completed in last 3 years and capable of CHO counting
- CBG measured at least twice daily for CGM calibration
- Capacity to follow the protocol and sign the informed consent
- Access to a personal computer/laptop

Participant exclusion criteria

- Severe episode of hypoglycaemia (requiring 3rd party assistance) in last 6 months
- Diabetic ketoacidosis in the last 6 months prior to enrolment
- Impaired awareness of hypoglycaemia (based on Gold score)
- Pregnant or planning pregnancy over time of study procedures
- Breastfeeding
- Enrolled in other clinical trials
- Active malignancy or being investigated for malignancy
- Suspected or diagnosed endocrinopathy like adrenal insufficiency, unstable thyroidopathy, endocrine tumour
- Gastroparesis
- Autonomic neuropathy
- Macrovascular complications (acute coronary syndrome, transient ischaemic attack, cerebrovascular event within the last 12 months prior to enrolment in the study)
- Visual impairment including unstable proliferative retinopathy
- Reduced manual dexterity
- Inpatient psychiatric treatment
- Abnormal renal function test results (calculated GFR <40 mL/min/1.73m2)
- Liver cirrhosis
- Not tributary to optimization to insulin therapy
- Abuse of alcohol or recreational drugs

- Oral steroids
- Regular use of the paracetamol, beta-blockers or any other medication that the investigator believes is a contraindication to the participant's participation.

Participant withdrawal criteria

- Loss of capacity to give informed consent
- The subject has a serious event related to study
- Cessation of MDI of insulin as usual care for T1DM
- Recurrent severe hypoglycaemia (requiring 3rd party assistance to manage)
- Diabetic ketoacidosis
- Pregnancy
- Terminal illness

Withdrawal will be immediate, and participants will be followed up in the appropriate out-patient diabetes clinic within 4 weeks of withdrawal.

Visit 1: Screening

- Participants will attend clinical research unit at 09:00
- Signed and dated informed consent
- Demographics (age, gender, date of birth, ethnicity)
- Clinical history, including current medicines, supplements and allergies
- Routine clinical examination including blood pressure, weight
- ECG
- Non-fasting venous blood taken for HbA1c, creatinine, lipids, liver function tests, full blood count and thyroid function test
- Urine pregnancy test in females of child bearing age
- Entry questionnaire completed by participant
- Structured education revision by a qualified healthcare professional (carbohydrate counting and hypoglycaemia management
- Real-time continuous glucose monitor (Dexcom G5) will be sited in the anterior abdominal wall according to manufacturer's instructions.
- Participants will be shown how to insert the sensor themselves, interpret the CGM data in real time and to set the hypo- and hyperglycaemia threshold alarms. The alarm threshold will be set at 4mmol/l and 11mmol/l and participants will be encouraged to keep it at those levels.
- Participants will use their existing capillary glucose meter for both glucose measurements and CGM calibration.
- Participants who do not own an iPhone will be provided with an iPhone 5 to monitor geolocation and ambient temperature.
- Participants will be provided with an Empatica E4 wristband to wear on either wrist according to manufacturer's instructions
- Participants will have the Empatica Real-time application installed on their smartphone device
- Participants will be required to have access to a personal computer/laptop and will be shown how to install and use the Empatica connect software.

- Participants will have the myTracks, Diasend and mySugr applications installed on their iPhone devices.
- Instructions to participants to ensure:
  - CGM calibration every 12 hours and sensor change after 7 days wear as per manufacturer's instructions.
  - All calibrations and CBG measurements will be undertaken by the participant.
  - Food diary (macronutrient composition - carbohydrate, fat and protein in grams), alcohol intake (units), intercurrent stress, and physical activity (duration and nature of exercise) to be logged in the mySugr smartphone application throughout the 6 weeks.
  - mySugr logbooks are exported as a spreadsheet on a weekly basis.
  - Empatica E4 wristband is connected to a personal computer (Windows or Mac) for an hour every day. This affords time for the wristband to charge and uploaded captured data using the proprietary Empatica connect software to a secure cloud-based platform.

Visit 2: Telephone consultation (Week 1)

- Investigator will communicate with participants 7 days following visit 1 to discuss
  - Any encountered technical issues
  - Uploaded CGM data reviewed remotely using the Diasend application
  - Uploaded Empatica E4 wristband data reviewed remotely using the E4 connect software
  - mySugr logbook spreadsheet sent to the investigator and reviewed remotely

Visit 3: Final visit at the end of 6 weeks

- Attend clinical research unit
- CGM data review with participant
- Empatica E4 and mySugr logbook review with participant
- Retrieve myTracks data from iPhone
- Empatica E4 and issued iPhone devices will be returned
- Exit questionnaire completed by participant

Usual care will be maintained for diabetes throughout the study. Participants will be offered support for their diabetes throughout the study. Participants will have the opportunity to call a physician for medical support and an engineer for technical support 24 hours a day.

Captured CGM and physiological data using the Dexcom glucose sensor and Empatica wristband respectively will be uploaded to a secure password encrypted cloud-based server. The Diasend and Empatica connect software will allow investigators to access this data remotely using encrypted passwords. Participants will be allocated a study number to ensure anonymity.

Each participant will have an individual patient ID number to enable labelling of samples sent to the Imperial College Healthcare Trust laboratories for immediate analysis.

Statistics

12 participants planned to be enrolled. This is not a randomised study and there are no comparison or control groups. Outcomes from the study are absolutes as described above.

Missing, unused, and spurious data will be assessed on an individual basis and may be ignored, withdrawn or the visit may be removed from the analysis with appropriate justification adjudicated by the Principal Investigator.

Table overview of activities (Phase 1)

Visit / Interaction no	1	2	3	Additional information
Consent	x			
Medical History taking	x			
Clinical examination (including weight and blood pressure)	x			
ECG	x			
Urine Pregnancy test	x			
Venous blood test	x			
Questionnaire(s)	x		x	
Insertion of CGM sensor	x			Participants will change the sensor every week at home throughout the study (24 weeks
Attaching Empatica E4 wirstband	x			
Issuing iPhone 5 and installing Diasend, mySugr and myTracks apps	x			
Empatica connect computer installation and training	x			
CGM review with participant		x	x	
Empatica E4 review with participant		x	x	
mySugr logbook review with participant		x	x	Participant will send excel of mySugr logbook to investigator
Removal of CGM			x	

Removal of Empatica E4 wristband			x	
Return issued iPhones and Empatica E4 wristbands			x	
Retrieving myTracks data			x	

3.0 Safety

Adverse Events

Definitions:
Adverse Event (AE): Any untoward medical occurrence in in participants, users or other persons whether or not related to the investigational medical device.

Serious Adverse Event (SAE): Adverse event that:
- Results in death, injury or permanent impairment to a body structure or a body function
- Is life-threatening
- Requires hospitalisation, or prolongation of existing inpatients' hospitalisation
- Results in persistent or significant disability or incapacity
- Results in foetal distress, foetal death or a congenital anomaly or birth defect

Planned hospitalization for pre-existing condition, or a procedure required by the Clinical Investigation Plan, without a serious deterioration in health, is not considered a serious adverse event.

Reporting Procedures

All adverse events will be reported. Depending on the nature of the event the reporting procedures below will be followed. Any questions concerning adverse event reporting will be directed to the Chief Investigator in the first instance.

Non-serious AEs
All such events, whether expected or not, will be recorded.

Serious AEs
A SAE form will be completed and faxed **or e-mailed** to the Chief Investigator within 24 hours. However, death and hospitalisations for elective treatment of a pre-existing condition do not need reporting as SAEs.

Reports of related and unexpected SAEs will be submitted within 15 days of the Chief Investigator becoming aware of the event, using the NRES SAE form for non-IMP studies. The Chief Investigator will notify the Sponsor and MHRA of all SAEs.

Contact details for reporting SAEs
Fax: 0207 594 2432, attention Prof Nick Oliver
nick.oliver@imperial.ac.uk
Please send SAE forms to:
Diabetes, Endocrinology and Metabolism Medicine
Imperial College
Room G3, Medical School Building
St Mary's Campus
Norfolk Place
London, W2 1PG
Tel: 0207 594 2460 (Mon to Fri 09.00 – 17.00)

Direct Access to Source Data/ Documents
The investigator(s)/institution(s) will permit study-related monitoring, audits, IRB/IEC review, and regulatory inspection(s), providing direct access to source data/ documents.

4.0 Regulatory Issues

Ethics Approval
The Chief Investigator has obtained approval from the Bloomsbury Research Ethics Committee and the HRA. The study will be conducted in accordance with the recommendations for physicians involved in research on human participants adopted by the 18th World Medical Assembly, Helsinki 1964 and later revisions.

Consent
Consent to enter the study will be sought from each participant only after a full explanation has been given, an information leaflet offered, and time allowed for consideration. Signed participant consent will be obtained. The right of the participant to refuse to participate without giving reasons will be respected. All participants are free to withdraw at any time from the protocol treatment without giving reasons and without prejudicing further treatment.

Confidentiality
The Chief Investigator will preserve the confidentiality of participants taking part in the study and is registered under the Data Protection Act.

All appropriate data and documentation related to study will be stored for 10 years and may be used to support other future research and may be shared anonymously with other researchers.

Indemnity
Imperial College London holds negligent harm and non-negligent harm insurance policies which apply to this study/ Imperial College Healthcare NHS Trust holds standard NHS Hospital Indemnity and insurance cover with NHS Litigation Authority for NHS Trusts in England, which apply to this study (delete as applicable)

Sponsor

Imperial College London will act as the main Sponsor for this study. Delegated responsibilities will be assigned to the NHS trusts taking part in this study.

Funding

This study is being funded by the Engineering and Physical Sciences Research Council (EPSRC) under grant agreement EP/P00993X/1

Audits

The study may be participant to inspection and audit by Imperial College London under their remit as sponsor and other regulatory bodies to ensure adherence to GCP and the NHS Research Governance Framework for Health and Social Care (2nd edition).

5.0 Study Management

The day-to-day management of the study will be co-ordinated by Chief Investigator at each recruiting site. Weekly research meetings and monthly data reviews will be chaired by the chief investigator or other senior researcher. Annual reports to the funder and sponsor will be written and submitted. The management team will meet at the conclusion of each phase of the study to review data and ensure that no events have occurred requiring progression to the study to be halted. The management team will meet again prior to commencing the next phase to ensure appropriate action is taken to mitigate risk of further events. The management team includes a lay member with diabetes and a consultant diabetologist not involved with the study.

6.0 Publication Policy

The study will be registered on the clinicaltrials.gov system and results will be disseminated by peer reviewed scientific journals, internal report, conference presentation and publication on websites. No identifiable personal data will be published. Anonymised quotes may be included in publications if the participant has given informed written consent. All anthropometry and personal clinical data will be expressed as mean/ median and spread of the population in the study. All participants will be informed of the results by letter at the conclusion of the study and details of any publications that arise from the study will be disseminated to participants.

References

1. NHS Digital 1 Trevelyan Square, Boar Lane, Leeds, LS1 6AE, United Kingdom. Quality and Outcomes Framework. http://content.digital.nhs.uk/qof. Accessed December 19, 2017.
2. NCD Risk Factor Collaboration (NCD-RisC). Worldwide trends in diabetes since 1980: a pooled analysis of 751 population-based studies with 4.4 million participants. *Lancet (London, England)*. 2016;387(10027):1513-1530. doi:10.1016/S0140-6736(16)00618-8.
3. Nathan DM, DCCT/EDIC Research Group for the DR. The diabetes control and complications trial/epidemiology of diabetes interventions and complications study at 30 years: overview. *Diabetes Care*. 2014;37(1):9-16. doi:10.2337/dc13-2112.
4. Group TDC and CTR. Hypoglycemia in the Diabetes Control and Complications Trial. The Diabetes Control and Complications Trial Research Group. *Diabetes*. 1997;46(2):271-286. doi:10.2337/DIAB.46.2.271.
5. Halawa I, Zelano J, Kumlien E. Hypoglycemia and risk of seizures: A retrospective cross-sectional study. *Seizure*. 2015;25:147-149. doi:10.1016/j.seizure.2014.10.005.
6. Sovik O, Thordarson H. Dead-in-bed syndrome in young diabetic patients. *Diabetes Care*. 1999;22 Suppl 2:B40-2. http://www.ncbi.nlm.nih.gov/pubmed/10097898. Accessed October 31, 2017.
7. Juvenile Diabetes Research Foundation Continuous Glucose Monitoring Study Group TJDRFCGMS, Bode B, Beck RW, et al. Sustained benefit of continuous glucose monitoring on A1C, glucose profiles, and hypoglycemia in adults with type 1 diabetes. *Diabetes Care*. 2009;32(11):2047-2049. doi:10.2337/dc09-0846.
8. Garg SK, Bookout TR, McFann KK, et al. Improved glycemic control in intensively treated adult subjects with type 1 diabetes using insulin guidance software. *Diabetes Technol Ther*. 2008;10(5):369-375. doi:10.1089/dia.2007.0303.
9. Lepore G, Dodesini AR, Nosari I, Scaranna C, Corsi A, Trevisan R. Bolus calculator improves long-term metabolic control and reduces glucose variability in pump-treated patients with Type 1 diabetes. *Nutr Metab Cardiovasc Dis*. 2012;22(8):e15-6. doi:10.1016/j.numecd.2012.04.001.
10. (UK) NCGC. *Type 1 Diabetes in Adults: Diagnosis and Management*. National Institute for Health and Care Excellence (UK); 2015. http://www.ncbi.nlm.nih.gov/pubmed/26334079. Accessed October 31, 2017.
